# Review and Prospect: Artificial Intelligence in Advanced Medical Imaging

**DOI:** 10.3389/fradi.2021.781868

**Published:** 2021-12-13

**Authors:** Shanshan Wang, Guohua Cao, Yan Wang, Shu Liao, Qian Wang, Jun Shi, Cheng Li, Dinggang Shen

**Affiliations:** ^1^Paul C. Lauterbur Research Center for Biomedical Imaging, Shenzhen Institute of Advanced Technology, Chinese Academy of Sciences (CAS), Shenzhen, China; ^2^Pengcheng Laboratrory, Shenzhen, China; ^3^School of Biomedical Engineering, ShanghaiTech University, Shanghai, China; ^4^School of Computer Science, Sichuan University, Chengdu, China; ^5^Shanghai United Imaging Intelligence Co., Ltd., Shanghai, China; ^6^School of Communication and Information Engineering, Shanghai University, Shanghai, China

**Keywords:** deep learning, magnetic resonance imaging, computed tomography, positron emission tomography, medical imaging reconstruction

## Abstract

Artificial intelligence (AI) as an emerging technology is gaining momentum in medical imaging. Recently, deep learning-based AI techniques have been actively investigated in medical imaging, and its potential applications range from data acquisition and image reconstruction to image analysis and understanding. In this review, we focus on the use of deep learning in image reconstruction for advanced medical imaging modalities including magnetic resonance imaging (MRI), computed tomography (CT), and positron emission tomography (PET). Particularly, recent deep learning-based methods for image reconstruction will be emphasized, in accordance with their methodology designs and performances in handling volumetric imaging data. It is expected that this review can help relevant researchers understand how to adapt AI for medical imaging and which advantages can be achieved with the assistance of AI.

## Introduction

Of all the advances in modern medicine, medical imaging is among the most remarkable developments. It allows us to see anatomical structures, organs, and biological processes unreachable by unaided eyes, providing tremendous opportunities for scientific research as well as disease diagnosis and treatment (1, 2). Different modalities such as magnetic resonance imaging (MRI) (3), computational tomography (CT) (4), and positron emission tomography (PET) (5) can provide versatile information, ranging from structure, morphology to physiological function. Specifically, MRI uses powerful magnetic fields, radio waves, and computers to produce details of anatomical structures and functions (6, 7). CT measures the linear attenuation coefficient of tissues inside each voxel element as an X-ray beam transmits through the body. PET measures changes in metabolic processes as well as other physiological activities by counting radioactive emissions of a biochemical metabolite labeled with radioactive material.

To better serve the clinical end-users, abundant studies have been conducted to optimize the scanning process, improve the imaging efficiency, and enhance the image quality of MRI/CT/PET (8, 9). Image reconstruction plays a significant role in this aspect. For MRI, its slow imaging speed has been a long-lasting bottleneck that seriously limits its wider applications in the clinic (10). Among different possible solutions, k-space undersampling has been identified as a highly effective approach to accelerate the scan (11, 12). Nevertheless, images generated from undersampled k-space data are subject to the low-quality issue, with possible loss of the important information related to disease diagnosis or treatment (13). Thus, high-quality image reconstruction from incomplete k-space data is critical. As for CT and PET, the main focus is to reconstruct high-quality images from deteriorated raw data caused by low-dose imaging demands (14, 15). Many efforts have been devoted to developing image reconstruction methods for MRI/CT/PET, among which deep learning-based methods have shown unprecedented successes (9, 14–16).

During the last decade, deep learning has been extensively applied to medical imaging to handle different problems, such as image reconstruction (17), image registration (18–20), image classification (21, 22), and lesion segmentation (23). Among these applications, image reconstruction is a primary step in the clinical workflow that has a huge impact on the downstream tasks of imaging-based analysis and decision making. Notice that different medical imaging modalities (MRI, CT, and PET) have their own unique imaging physics and principles, and thus numerous deep learning-based methods have been proposed to accomplish respective reconstruction tasks (9, 11, 14, 15). For MRI, existing works have achieved impressive achievement to balance imaging efficiency and imaging quality (9, 11). Similarly, promising results have also been achieved for CT and PET image reconstruction (14, 15). However, current progress is still preliminary for deep learning-based image reconstruction in real applications, and more efforts are needed to make this technology mature enough for wide real-world clinical applications. Thus, it is the right time to review existing works to help beginners as well as non-specialists better understand this relatively new technique and promote more follow-up investigations and applications.

The remainder of this review paper is organized as follows. In section Overall Workflow of Deep Learning-Based Reconstruction, we demonstrate the overall workflow of deep learning-based reconstruction, by briefly introducing the basics of deep learning relevant to the reconstruction task, the purpose of image reconstruction, and the workflow of deep learning-based reconstruction. Detailed technical developments of deep learning-based reconstruction are introduced in section Technical Developments of Deep Learning-Based Reconstruction. Section Clinical Applications and Current Achievements reviews current clinical applications and achievements, followed by descriptions of key challenges and opportunities in section Challenges and Opportunities. Finally, section Conclusion concludes the paper.

## Overall Workflow of Deep Learning-Based Reconstruction

### Basics of Deep Learning

Artificial intelligence (AI) refers to the ability of a machine to simulate human intelligence by thinking and acting like humans (24). Deep learning is a sub-discipline of AI, which specifically addresses various tasks through building deep neural networks (DNNs) (25). Different abstract levels of representations are extracted with multi-layer networks which enable the learning of complex functions. When inputs are images, the low-level features usually represent edges and contours in the images, whereas the high-level features are commonly semantic features (26). One key characteristic for deep learning is that all the parameters for feature extraction are learned automatically with the provided data samples, which can be better self-optimized to specific problems compared to the use of manual feature engineering approaches (26, 27).

Supervised learning, unsupervised learning, and reinforcement learning are the three major paradigms for deep learning (28–31). Supervised learning requires paired data samples for the inputs and the expected outputs (28). Model optimization is performed by minimizing loss functions that are calculated to measure the difference between model outputs and ground truth. In unsupervised learning, only input data samples are provided, and certain assumptions of the data have to be made and then the corresponding model constraints are enforced to facilitate the model learning (29). In reinforcement learning, an algorithm is referred to as an agent. Then, the agent takes an action to change its state, and, at the same time, a reward or penalty is assigned. Different from supervised learning, the training data of reinforcement learning provide only an indication of whether an action is correct or not. The overall goal of reinforcement learning is to achieve the maximum reward over time by learning a policy for the agent to choose proper actions for any given states (31). Most medical image reconstruction models are based on supervised learning or unsupervised learning, while reinforcement learning is less frequently utilized.

### Deep Learning-Based Image Reconstruction

#### MRI

MRI reconstruction aims to generate high-quality images from sampled k-space data. Conventional reconstruction methods (i.e., Fourier transform) require the scanning process to follow the Nyquist sampling theory. Thus, to obtain high-quality images, the sampling frequency should be high enough, which unfortunately makes the scanning process very time-consuming. On the other hand, undersampling, which breaks the Nyquist sampling theory, leads to imperfect MR image reconstruction if using conventional reconstruction methods. To this end, compressed sensing (CS) MRI (CS-MRI) has been proposed by introducing CS theory to reconstruct MR images with significantly fewer measurements than those required by traditional Nyquist sampling theory (32). CS-MRI accomplishes the reconstruction task mainly by exploiting the sparsity of MRI, since most MR images are sparse after transformed into an appropriate domain (32), such as using total variation (33) and wavelet transformation (34).

Despite the successes achieved, CS-MRI still has limited performance because of using manually-designed methods to exploit the sparsity in MRI. By contrast, deep learning-based image reconstruction for MRI can automatically and fully exploit the available data information and recover the lost information under the guidance of certain prior knowledge. Deep learning was first introduced to MR image reconstruction in 2016 by Wang et al. (35). In their work, a three-layer neural network was built to automatically learn the mapping between low-quality and high-quality images (35). Following this work, a series of studies have been published, aiming to build more sophisticated, robust, and optimized deep learning models for MR image reconstruction (36–39).

Existing deep learning-based MR image reconstruction methods can be classified into two major categories, (1) model-based methods and (2) data-driven methods. Model-based methods reconstruct high-quality MR images via solving certain optimization algorithms and utilizing neural network modules to represent the reconstruction steps of the solution. Typical optimization algorithms include alternating direction method of multipliers (ADMM) algorithm (40), iterative shrinkage-thresholding algorithm (ISTA) (41), and primal-dual hybrid gradient (PDHG) algorithm (42). Data-driven methods are the end-to-end approaches that rely on DNNs with large capacities to learn non-linear reconstruction processes. Example models include U-Net (36), residual network (ResNet) (43), and generative adversarial networks (GAN) (44). Model-based methods are more interpretable as the network blocks can correspond to the algorithm solutions, and data-driven methods are more effective in data exploitation. Overall, deep learning-based MR image reconstruction methods have dominated the current research field, with promising performance.

#### CT

In CT, image reconstruction aims to transform the sensor data, which basically reflects line integrals of the object, to an image representing the object. Until recently, most CT reconstruction methods can be classified as either analytic reconstruction or iterative reconstruction. Analytic reconstruction is based on the mathematical inverse of the forward model of an imaging process, which could either be mathematically derived or numerically modeled after the design of the CT imaging device and the knowledge about how it generates sensor data. A typical example of analytic reconstruction in CT is filtered back-projection (FBP) (45). Iterative reconstruction is based on a numerical forward model combined with a feedback loop (46–51). In the feedback loop, the error between the calculated sensor dataset and the measured sensor dataset is back-transformed to the image domain to update the current image estimation. This process is repeated until the error reaches a small threshold and the optimum image solution is obtained. Iterative reconstruction has been widely used in CT because the measurements are typically noisy or a mathematical inverse is unknown or computationally challenging. Examples of iterative reconstruction in CT include the algebraic reconstruction technique (ART) (52) and the simultaneous algebraic reconstruction technique (SART) (46). Iterative reconstruction usually outperforms analytic reconstruction in terms of the quality of reconstructed images, because iterative reconstruction relies on a more improved forward model and has the ability to bring in various types of external prior information to expand the information available during reconstruction.

Very recently, a third type of CT reconstruction method – deep learning based reconstruction – was introduced. Deep learning reconstruction was first introduced to CT in 2016, when Kang et al. used a deep learning reconstruction approach at the 2016 Low-Dose X-ray CT Grand Challenge [organized by the American Association of Physicists in Medicine (AAPM)] (53), and, in parallel, when Chen et al. introduced a similar convolutional neural network (CNN) for low-dose CT denoising (54). The successful demonstration of CNN reconstruction in low-dose CT has inspired many other deep learning reconstruction research. For example, a combination of a CNN with the Normalized Metal Artifact Reduction (NMAR) algorithm for CT metal artifact reduction (55), a combination of DenseNet and Deconvolution Network (DD-Net) for sparse-view CT (56), Super-Resolution Convolutional Neural Network (SRCNN) for CT super-resolution (57), and so on.

Deep learning reconstruction does not require an explicit physical imaging model. Instead, deep learning reconstruction can build its own model from a large amount of training data, which becomes more and more readily available due to the wide use of medical imaging in modern healthcare. With larger and more representative training datasets, deep learning reconstruction has the potential to outperform both analytic reconstruction and iterative reconstruction. With unsupervised learning or self-supervised learning, it has been hypothesized that the integration of imaging physics within the machine learning pipeline may further improve the reconstruction quality. For example, a self-supervised and hybrid CT super-resolution model that integrates the advantages of both deep learning network and imaging physics has been just published very recently (51).

#### PET

Similarly, PET reconstruction aims to generate diagnostic quality images from measurement data. The conventional PET reconstruction methods can be broadly classified into two categories, i.e., (1) analytic (58, 59) and (2) iterative PET reconstruction methods (60, 61). The analytic PET reconstruction methods provide a straightforward mathematical solution for image formation, a typical example of which is the filtered-back projection (FBP). In contrast, based on a more accurate description of the imaging process, iterative methods produce a more complex mathematical solution that requires multiple steps to reach an image. Since it can take into account the noise patterns in the observations and use more realistic models of the system, the iterative methods provide improvements over the analytical methods. The classical iterative methods include Maximum Likelihood-Expectation Maximization (ML-EM) (60) and Ordered Subsets Expectation Maximization (OSEM) (61).

Recently, numerous learning-based methods have also been developed for PET reconstruction, such as random forest (62), sparse representation (SR) (63), and multi-level Canonical Correlation Analysis (mCCA) scheme (64). Yet, these traditional machine learning methods often require complex feature engineering, which largely limits the practicability and also results in suboptimal reconstruction quality. To address this limitation, deep learning was first introduced to PET reconstruction in 2017 by Xiang et al. (65). The authors proposed a deep CNN model, followed by an auto-context strategy, to estimate standard-dose PET images directly from both the low-dose PET and the corresponding MR images, without the need for handcrafted features. Encouraged by the great success of this work, a series of deep learning-based methods have been developed and successfully applied to various scenarios of PET reconstruction (58–61, 66, 67). In addition, the combination of the conventional iterative reconstruction framework and the deep learning-based method has provided some new approaches for PET reconstruction (14, 68, 69). For instance, Gong et al. (14) used the existing inter-patient information *via* a deep neural network to further improve the quality of the reconstructed PET image. Furthermore, with the introduction and development of new deep learning models such as GAN, more efforts applying new techniques have been continuously conducted for superior PET reconstruction performance (70–72).

### Training and Testing Workflow

The image reconstruction framework typically includes an input, a reconstruction model, and an output. Traditionally, the input is a sensor-domain raw data, i.e., sinogram in CT. With deep learning-based reconstruction, the sensor data can be first reconstructed using an analytic reconstruction model to provide a low-quality image, and then this low-quality image is fed into the DNN model to generate the corresponding high-quality image. For MRI, the input and output data pair can be either in k-space or image space. Note, to build a deep learning-based reconstruction framework, two steps, namely model training and model testing, are included, as detailed below.

Model training is performed on the provided training samples to optimize the model parameters. During the model training, the loss between the model-generated outputs and the provided training samples is calculated and back-propagated to optimize the model parameters. The model parameters are updated to minimize this loss. Model training proceeds in a data batch mode. Training is stopped after the model is converged to a certain point, or after reaching a pre-selected number of epochs. To avoid the overfitting issue, data augmentation is commonly utilized. Frequently utilized data augmentation methods include affine transformations and Gaussian noise addition. In a deep learning model, there are usually some hyper-parameters (such as batch size, learning rate, etc.) that need to be adjusted manually or automatically, i.e., using an additional validation set, to improve the model performance.

With the optimized model, testing can be performed. To comprehensively evaluate the model performance, testing with data different from the training/validation data should be conducted. For example, validating and testing data from different centers collected with different machines are often considered to make the model robust enough in real-world applications.

## Technical Developments of Deep Learning-Based Reconstruction

This section will review various deep learning reconstruction methods developed for MRI, CT, and PET, with typical methods summarized in Table 1. We will present technical aspects and performance characterization of deep learning reconstruction. Technical aspects will include data preparation, network architecture design, loss function, and settings or requirements for training.

**Table 1:** Representative works on deep learning-based MRI/CT/PET image reconstruction.

**Modality**	**Task description**	**Network architecture**	**Loss function**	**Dataset**	**Evaluation metrics**	**Reference**
MRI	Directly learning the transformation from sensor-space data to image	MLP	Simple squared loss and additional L1-norm penalty	ImageNet database, MGH-USC HCP public database	SNR, RMSE	(37)
MRI	k-space to k-space reconstruction	UNet	L2 loss	Knee k-space dataset, MGH-USC HCP public database	NMSE, PSNR, SSIM	(36)
MRI	Reconstruction with proposed complex convolution operations	ResNet	Mean absolute error (MAE)	Brain dataset, Knee dataset	PSNR, SSIM	(43)
MRI	Reconstructing real-valued and complex-valued MRI data	GAN	Cyclic data consistency loss	IXI database, Data Science Bowl challenge, Knee dataset	PSNR, SSIM, NRMSE	(44)
MRI	Fast and high-quality reconstruction by combining various loss functions	GAN	Content loss, Image domain and frequency domain MSE loss, Perceptual VGG loss	MICCAI 2013 grand challenge dataset, Pathological MRI images	NMSE, PSNR, SSIM	(52)
MRI	Infusing motion information into the modeling process with deep neural networks for enhanced dynamic MRI reconstruction quality	Recurrent neural network (MODRN, Motion-guided Dynamic Reconstruction Network)	L1 loss	Private short-axis cardiac data (21 normal subjects and 3 dyssynchrony disease patients)	NMSE, PSNR, SSIM	(73)
MRI	Reconstruction with both k-space and spatial prior knowledge integrated via multi-supervised network training	CNN	L2 loss	Private cardiac MR data	MSE, PSNR, SSIM	(74)
MRI	Improving MRI reconstruction accuracy and computational speed with a CS-based model	Model-based (Alternating direction method of multipliers algorithm)	NMSE	Brain and chest MR images	NMSE, PSNR, Test time	(40)
MRI	Fast and high-quality reconstruction of clinical accelerated multi-coil MR data	Model-based (Variational network, unrolling iteration)	MSE	Clinical knee dataset	SSIM, NRMSE	(38)
MRI	Deriving deep architectures for inverse problems with the arbitrary structure	Model-based (recursive framework alternating between denoising block and data-consistency layer)	MSE	Brain MR dataset from five volunteers	PSNR, Time	(75)
MRI	Fast parallel MR imaging by exploring both spatial redundancy and multi-coil correlations	Model-based (split Bregman iterative algorithm)	MSE	Private 2D multichannel MR brain dataset	NMSE, PSNR, SSIM	(76)
MRI	Self-supervised deep learning MRI reconstruction by dividing sub-sampled data points into two sets with one for data consistency and another for loss calculation	Model-based (regularized iterative algorithm between data consistency and a regularizer solved by the variable-splitting and quadratic relaxation method)	Normalized L1-L2 loss	Knee MR data from fastMRI initiative database	NMSE, SSIM	(77)
MRI	Accelerate and improve multishot diffusion-weighted MRI reconstruction by combining unrolled network with deep CNNs	Model-based and UNet (recurrences of model-based gradient updates (shotlocally low-rank) and neural networks	L1 loss	Private brain (14 scans from 8 volunteers) and breast (6 scans from 6 volunteers) MR data	NMSE, PSNR, SSIM	(78)
CT	Using U-Net and its variants for recovery of high-frequency edges in sparse-view CT in the image domain	Dual frame and tight frame U-Nets	Pixel-wise soft-max combined with cross entropy function (the original U-net loss function)	10 patient CT scan data from the 2016 AAPM Low Dose CT Grand Challenge Dataset	NMSE, PSNR, SSIM	(56)
CT	General sparse-view CT image reconstruction	DenseNet combined with deconvolution	Weighted loss between MSE and MS-SSIM	3,059 clinical CT images from the TCIA database	MSE, SSIM, Haralick texture features	(15)
CT	CT super-resolution	Modified U-net	L2 loss	7,670 CT slices	NRMSE, PSNR	(57)
CT	Low-dose CT for mapping low-dose images to normal-dose images; CT image denoising	Residual encoder-decoder CNN (RED-CNN)	MSE loss	7,015 normal-dose CT images from the NBIA dataset and simulated low-dose CT images	RMSE, PSNR, SSIM	(54)
CT	CT image denoising	Framelet-based wavelet residual network	Pixel-wise soft-max combined with cross entropy function (the original U-net loss function)	10 patient CT scan data from the 2016 AAPM Low Dose CT Grand Challenge Dataset	RMSE, PSNR, SSIM	(79)
CT	Sparse-view CT image reconstruction	U-net with skip connection for residual learning	Pixel-wise soft-max combined with cross entropy function (the original U-net loss function)	The 2016 AAPM Low Dose CT Grand Challenge Dataset, plus 500 simulated images and 377 experimental sinograms	SNR	(80)
CT	CT image denoising in low-dose CT	GAN network, consisting of a Generator CNN and a Discriminator CNN	binary cross-entropy, L2 loss	5 low-dose and 5 corresponding routine-dose CT scans of a phantom, and 28 cardiac CT scans from patients	SNR, PSNR	(81)
CT	CT image denoising in low-dose CT	GAN network with Wasserstein distance and perceptual loss (WGAN)	Wasserstein distance based adversarial loss, VGG perceptual loss	10 patient CT scan data from the 2016 AAPM Low Dose CT Grand Challenge Dataset	PSNR, SSIM	(82)
CT	CT super-resolution	GAN-CIRCLE	Adversarial loss, cycle consistency loss, identity loss, joint sparsifying transform loss	Micro-CT dataset from 25 tibia specimen, and the 2016 AAPM Low Dose CT Grand Challenge Dataset	PSNR, SSIM, IFC	(83)
CT	To ensure data consistency even in worst-case scenario, and to guarantee the convergence of a non-convex CT reconstruction problem	Specially designed method that replaces the projector in a projected gradient descent with a CNN, and uses the CNN in the feedback loop to recursively project the result onto the sensor domain	Data consistency loss	500 lower-lung CT images from the 2016 AAPM Low Dose CT Grand Challenge Dataset, and 377 micro-CT slice images of a rat brain	SNR, SSIM	(84)
CT	CT Super-resolution	Self-supervised SADIR-net (super-resolution and deblur based iterative reconstruction), which is a hybrid between deep learning network and imaging physics	Joint loss function combining L2-norm with SSIM	47 clinical CT scans from TCIA database; custom-acquired Catphan^700^ phantom CT sensor data	MTF, RMSE, SSIM, IFC	(51)
PET	Incorporating the neural network into the iterative PET reconstruction framework for PET denoising	UNet with residual learning	Augmented Lagrangian format, L2 loss	19 XCAT phantoms; 6 lung patient data	CR, STD	(14)
PET	Standard-dose PET reconstruction from low-dose PET	Noise-Aware Dual Res-UNet	Dice loss, Binary cross entropy loss, General and adaptive robust loss, SSIM loss	10 subjects referred for whole-body FDG-18 PET/CT scan on a GE Discovery 710 scanner	PSNR, SSIM	(85)
PET	Using patients' own prior information for PET reconstruction	3D UNet	MSE	Phantom and real brain data	CRC, STD	(69)
PET	PET reconstruction from projections data	ANN	MSE	Simulated data	NMSE	(86)
PET	Using multilayer perceptron (MLP) to enhance MAP reconstructed PET images	MLP with backpropagation	Least squares loss	PET phantom images, two patient PET imaging datasets	NMSE, NSD, Contrast	(87)
PET	Ultra-low-dose PET reconstruction	ResNet	L1 loss, SSIM, MS-SSIM	9 PET/MRI images from patients with glioblastoma (GBM)	PSNR, SSIM, NRMSE	(66)
PET	Using dilated convolutions for recovering full-count PET images from low-count PET images	UNet with dilated convolution	L1 loss	35 PET data extracted from an IRB approved psychiatric study	MAPE, PSNR, SSIM	(88)
PET	Reconstruction of PET image from sinogram data	CNN	VGG, MAE, MS-SSIM	Whole-body PET studies:40 patients for training, 4 for validation, and 10 for testing	SNR, Bias, MAE, MS-SSIM	(89)
PET	Anatomy-aided PET image reconstruction	3D CNN	L2 loss	Simulation study: 20 XCAT51 phantoms real patients studies: 6 hybrid lesion patients, 6 lung cancer patients	CR, STD	(90)
PET	Using a deep learning prior for iterative PET reconstruction	DnCNN + local linear fitting (LLF)	L2 loss	27 control subjects and clinical patients	Bias and standard deviation; NRMSE; SSIM	(68)
PET	Reconstruction of PET image from sinogram data	GAN	Adversarial loss, L1 loss	Simulated data of the three phantoms using Monte Carlo simulations, including Zubal thorax phantom with 64Cu-ATSM, Hoffman brain phantom with 18F-FDG and Zubal brain phantom with 11C-Acet ate	Bias, Variance	(71)
PET	Reconstruction of PET images from sinogram data	GAN	MSE, Relativistic Average LS adversarial loss	Human brain PET dataset with nine subjects	Bias, Variance, PSNR, SSIM	(70)
PET	Low-dose PET image denoising	CycleWGAN	Adversarial loss, Cycle-consistency loss, Identity loss	Eighteen patients with biopsy-proven primary lung cancer or patients with suspicious radiological abnormalities	NRMSE, PSNR, SSIM, *SUV*_*mean*_ and *SUV*_max_	(72)

### Data Preparation

When applying deep learning to medical imaging, normally three datasets are in need, namely training, validation, and testing datasets. The training dataset is used to train a neural network that is monitored by the validation dataset to avoid overfitting or underfitting. The testing dataset is to evaluate whether the deep learning models can perform well for the real application scenarios. The datasets should include ground-truth images for supervised learning. While for unsupervised learning, no ground-truth information is needed.

For MRI, different types of datasets have been collected and experimented with for various applications. According to the target region dynamic characteristics, there are static MRI and dynamic MRI. Static MRI is applicable when the imaging target changes slowly with time, such as the knee (36, 38) and the brain (37). Dynamic MRI is often required when the target moves fast, such as cardiac MRI (74, 91). Based on the number of coils utilized to collect the data, MRI datasets can be classified into single-channel MRI (92) and multi-channel MRI (43, 76, 93). When different imaging parameters are used, multi-parametric MRI data are collected to better characterize the physical and physiological properties of the imaging object (94). Besides, quantitative MRI is also available, which can measure tissue-specific parameters (95)^1^.

For CT, depending on the goal of network training, various public datasets are available for DNN model training when developing deep learning reconstruction methods. Some datasets are curated for image noise reduction. For example, the Mayo Clinic Low-Dose X-ray CT datasets for the Low Dose CT Grand Challenge organized by the AAPM (54) have clinical CT images acquired at the full-dose level and the corresponding simulated CT images at the quarter-dose level. This Mayo Clinic dataset can be useful for training deep learning models to reduce CT image noise and hence optimize the dose efficiency. Other datasets are curated toward specific diseases or conditions. For example, The Cancer Imaging Archive (TCIA) hosts a large archive of medical CT images of cancer accessible for public download. Noticeably, in the last year, because CT has been successfully proven to be a rapid triaging tool in patients with moderate to severe COVID symptoms in a resource-constrained environment where COVID-19 is highly prevalent (96), we now have abundant publicly-available COVID CT datasets available today. Two particular COVID CT datasets could be useful for training deep learning models. One is the BIMCV-COVID-19+ (97), a large dataset from the Valencian Region Medical Image Bank earlier in the pandemic period, and another is the RSNA International COVID-19 Open Radiology Database (RICORD), which is an ongoing international effort in curating potentially the largest international COVID-19 CT dataset.

For PET, the datasets mainly include static PET (98–100) and dynamic PET (101–104) based on data types. On the other hand, according to the number of tracers imaged in a single scan, the datasets can be classified as single-tracer PET (105), dual-tracer PET (106, 107), and multi-tracer PET (108). When it comes to the injected tracer dose level, the datasets can also be broadly categorized as low-dose PET (L-PET) and full-dose PET (F-PET) (65, 67, 88). Although the use of real PET data in studies is more clinically relevant, these real data are often difficult to obtain due to various factors. Therefore, simulated phantom data is becoming a popular alternative in research works (68, 88, 109, 110).

### Network Architecture

The neural network architectures employed for different tomographic imaging tasks share some similar properties. The most frequently used architectures include multilayer perceptron (MLP), U-Net, generative adversarial networks (GAN), ResNet, etc. Here, we introduce these typical network architectures.

#### MLP

The MLP, which is an artificial neural network (ANN) with all layers fully-connected, can map sets of input data into a set of desired outputs. In the past decades, researchers have worked on exploiting MLP in medical image analysis. For example, a multilayer perceptron was proposed for accelerated parallel MRI (111). Zhu et al. (37) proposed an MLP-based manifold learning framework to emulate the fast-Fourier transform and learn an end-to-end mapping between k-space data and image domains and achieve the purpose of acceleration. For PET, MLP was also employed for simple low-resolution PET reconstruction (86). Furthermore, Yang et al. (87) developed an MLP-based framework to enhance the maximum a posteriori (MAP) reconstructed PET images, which constructs a highly non-linear and spatial-varying mapping between the MAP reconstructed image patches and the corresponding enhanced image patches.

#### U-Net

U-net consists of an encoder structure and a decoder structure, which was originally designed for biomedical image segmentation (112, 113). The encoder gradually down samples the input images to extract image features with different levels of semantic information. The decoder receives the features from the encoder and recovers the feature map resolution step-by-step to generate the outputs, which are often the same size as the inputs and can then be treated as the reconstructed images. Skip connections between the encoder and the decoder are introduced to improve the localization accuracy during decoding.

For MRI, Ye et al. (114) used deep residual learning to accelerate MRI. The proposed deep residual learning network is composed of two separately trained amplitude and phase difference networks, which can successfully learn and remove aliasing artifacts. Furthermore, Ye et al. also proposed a U-Net-based domain adaptation architecture for radial k-space undersampled MR (115), and a fully data-driven deep learning algorithm for k-space interpolation (36). These methods have been successfully applied to MR image reconstruction, and have achieved better results than the classic CS method. Duan et al. (116) proposed a fast and accurate deep learning reconstruction method for human lung gas MRI, which consists of coarse-to-fine nets (C-net and F-net) based on U-Net. The proposed deep learning method can better reconstruct the human lung gas MR images acquired from highly undersampled k-space compared with the traditional CS-MRI. Hyun et al. (117) proposed an under sampling MRI reconstruction method using U-Net, which shows excellent performance and can generate high-quality MR images with a small amount of data.

For CT, U-net and its variants have also been successfully applied to solve various problems in CT reconstruction, including sparse-view CT reconstruction, artifact reduction, noise suppression, and CT super-resolution, etc. For sparse-view CT reconstruction, which can reduce radiation dose and accelerate scanning speed, Han et al. (56) achieved better reconstruction performance by framing U-Net via deep convolutional framelets. Also, for sparse-view CT reconstruction, Kofler et al. (118) proposed a cascade of U-nets and data consistency layers, and Zhang et al. (15) developed DD-Net by combining DenseNet and deconvolution and arranging them in a network topology similar to U-Net. For the purpose of CT artifact reduction, Zhang et al. (55) tried U-net and found promising results of U-net in reducing global and local CT artifacts. To reduce noise in low-dose CT images, Liu et al. (119) adopted stacked denoising autoencoders to suppress noise and recover structure details. For CT super-resolution, Park et al. (57) used a modified U-net to learn an end-to-end mapping between low-resolution and high-resolution CT images.

For PET, U-net is also a commonly used framework in many PET reconstruction works (14, 69, 86, 120–122). Gong et al. (14) designed an iterative reconstruction framework that combines the U-net structure and the residual network for PET denoising by utilizing dynamic data of prior patients. Taking the noise level of low-count PET into account, Xiang et al. (85) developed a noise-aware dual Res-UNet (NADRU) framework for low-dose PET reconstruction. The proposed method first identified an attention map indicating the location of high-intensity noise in the low-dose PET images. Then, the noise attention map was incorporated with the original image for high-quality PET reconstruction. In addition to reconstructing high-quality images within PET, many efforts have also been made to reconstruct PET from other modalities. For example, Sikka et al. (121) adopted a 3D U-Net architecture to estimate PET from MRI images. By considering non-local and non-linear correlations, the proposed method showed a significant improvement in the diagnostic accuracy of Alzheimer's disease. Employing a modified 3D U-net as the network structure, Gong et al. (69) designed an iterative reconstruction framework that incorporates the personalized deep neural network to generate PET data from a patient's own MRI prior image(s). Furthermore, Cui et al. (122) utilized CT/MR prior information to perform PET denoising based on a modified 3D U-net structure in an unsupervised manner.

#### ResNet

ResNet is proposed to solve the difficulty of training very deep CNNs and avoid model performance degradation (123). The core idea of ResNet lies in residual learning, which is based on the assumption that it is easier to optimize the residual mapping than to optimize the original and unreferenced mapping (123). With the success of residual learning, the ResNet has also been widely used in medical image reconstruction.

For MRI, Shi et al. (124, 125) proposed a residual-learning-based MR image super-resolution reconstruction network. The network can improve image reconstruction performance using both global residual learning (GRL) and local residual learning (LRL). Wang et al. (43) proposed a new framework Deepcomplex MRI using a deep residual CNN for parallel imaging. It considers the correlation between the real and imaginary parts of MR complex images and achieved better results than real-value networks. Li et al. (126) designed a deep ResNet using variable density spiral trajectory to accelerate fMRI reconstruction. The proposed deep ResNet consists of various residual blocks. Du et al. (127) proposed a residual CNN for reconstructing single anisotropic 3D MR images based on residual learning. The residual CNN with long and short skip connections can effectively recover uncollected high-frequency details of MR images.

For CT, ResNet or more generally residual learning has also been demonstrated its effectiveness in CT reconstruction, particularly in noise suppression and artifact reduction. Chen et al. (128) developed a residual encoder-decoder CNN (RED-CNN) for low-dose CT. RED-CNN combines autoencoder, deconvolution network, and shortcut connections. It can effectively suppress noise, preserve structure details, and enhance lesion detection. For CT image denoising, Kang et al. (79) proposed a wavelet residual network based on a deep convolutional framelet and achieved better performance compared to their earlier algorithm using directional deep convolutional-wavelet neural network (53). To reduce the sparse-view CT artifact, Dong et al. (129) proposed a residual deep learning CNN to interpolate the sinogram of sparse-view micro-CT, and the deep learning interpolated sinogram was FBP-reconstructed into high-quality images. Also for sparse-view CT, Jin et al. (81) proposed FBPConvNet, which first reconstructs sparse-view CT sinogram with FBP and then improves the FBP-reconstructed image using a modified U-net with the addition of residual learning.

For PET, residual learning is also employed in the reconstruction task. In order to effectively restore the low-dose PET images to the standard-dose quality, Xu et al. (66) proposed an encoder-decoder residual deep network, in which residual learning and skip connections were adopted for learning the difference between standard-dose and low-dose PET images. Similarly, Spuhler et al. (88) designed a novel multiscale dilated CNN approach to predict full-count PET images from low-count images. The proposed method integrated the residual learning to capture the difference of low-count and full-count PET images and enhance the convergence of the network. The experiments of these studies showed that residual learning was beneficial for high-quality PET reconstruction. Moreover, in Chen et al. (54), a deep learning-based framework with low-count PET and multimodal MRI as inputs was presented for diagnostic-quality PET image synthesis through residual learning.

#### GAN

GAN (130), as one of the most popular generative models in deep learning, has demonstrated its superior performance in many computer vision tasks and attracted growing interest in medical image reconstruction.

For MRI, Yang et al. (52) proposed De-Aliasing GAN (DAGAN) for fast compressed sensing MRI reconstruction. The authors designed a refinement learning method to stabilize the U-Net-based generator. In order to better preserve texture and edge information, DAGAN combines adversarial loss and innovative content loss in the image reconstruction process and takes into account the frequency information at the same time. The reconstruction result of DAGAN is better than the traditional CS-MRI algorithm. Quan et al. (44) proposed an improved model, RefineGAN, based on fully residual convolutional autoencoder and GANs for fast and accurate CS-MRI reconstruction. It can perform faithful interpolation for a given undersampled k-space data by employing a deeper generator and discriminator with cyclic data consistency loss. RefineGAN outperforms the state-of-the-art CS-MRI reconstruction algorithms in terms of both image quality and running time. Mardani et al. (131) proposed a novel CS framework based on LSGAN and pixel-wised l1/l2 loss for MRI reconstruction, namely GANCS. GANCS can reconstruct higher quality images with improved fine texture details compared to existing methods.

For CT, Wolterink et al. (81) used a GAN network that consists of a Generator CNN and a Discriminator CNN to reduce the noise level in CT images. They produced better images for more accurate coronary calcium quantification. Similarly, for the purpose of image denoising in low-dose CT, Yang et al. (82) modified the original GAN network by using the Wasserstein distance, instead of the Jensen-Shannon (JS) divergence, to compare data distributions. The Wasserstein distance is combined with the well-known pre-trained VGG-19 network (132) to build a joint loss function. This modified GAN network also achieved promising results in image denoising. For the purpose of CT super-resolution, You et al. (83) developed a GAN network constrained by the identical, residual, and cycle learning ensemble (GAN-CIRCLE). GAN-CIRCLE incorporates deep CNN, residual learning, and network-in-network techniques for feature extraction and restoration, and employed a cycle Wasserstein regression adversarial training framework. It is noted that many GAN networks also employed the technique of residual learning in their architectures.

For PET, Liu et al. (71) employed a conditional GAN (cGAN) framework to learn the mapping from sinogram data to reconstructed PET images directly. Inspired by the promising results achieved by cGAN, the authors further presented an end-to-end model for PET reconstruction, which adopts two coupled networks to sequentially denoise low dose sinogram and reconstruct activity map (70). Zhou et al. (72) designed a cycle Wasserstein regression adversarial model (CycleWGAN) using Wasserstein distance, instead of JS divergence and cycle-loss, to boost the low-dose PET image quality, which shows the superior performance of Wasserstein distance in effectively preserving the edge information. To reduce the loss of contextual information, Wang et al. (133) developed a concatenated 3D cGAN for high-quality PET image estimation from low count PET. Considering the various contributions of different image locations and the complementary information in different modalities, they further proposed an auto-context-based locality adaptive GANs (LA-GANs) (67) model to reconstruct the full count PET image from both the low count PET and the accompanying multimodal MRI images. Besides, many other works also attempted to reconstruct PET images from other modality information in consideration of the expensive cost of PET imaging and the hazards of radiation exposure. Ben-Cohen et al. (134) proposed to generate simulated PET images from given CT data without manually annotated labels. They first adopted FCN to generate an initial PET-like image and then employed cGAN to refine the FCN output so that the synthesized image could be more realistic. Based on 3D GAN, Yaakub et al. (135) designed a two-stage approach to predict accurate PET images from T1-weighted MRI scans. It is worth noting that many GAN-based models have also introduced residual learning to further improve the reconstruction performance (136, 137).

#### Modality-Specific Module Design

To improve the reconstruction accuracy or enhance the reliability of the reconstruction results, special network modules are usually designed taking the specific properties of different imaging modalities into consideration.

For MRI, in addition to modules utilized by every model, including the convolutional layers, the normalization layers, and the activation layers, there is commonly a data consistency layer to guarantee that the data on scanned points are correct (138). According to the data acquisition process of MRI, undersampling happens in the k-space by neglecting a certain portion of data points. Therefore, theoretically, on the scanned data points, the reconstruction results should be consistent with the acquisitions. With the data consistency layer, the reconstruction is forced to be correct on these sampling points and the reconstruction of unscanned data points is accordingly improved. Besides, because the data acquisition of MRI proceeds in a different domain (k-space) from the image domain, reconstruction can be performed in individual domains (38, 139) or cross-domains (74, 140). Furthermore, complex-valued neural networks are proposed to specifically process the complex-valued MR data (43, 141, 142).

For CT, although the reconstruction results from most reported deep learning algorithms are so far remarkable in terms of image quality, there is still some concern about whether those reconstruction results can be trusted, especially in real-world applications of diagnostic imaging. One main limitation of those deep learning algorithms is that they seldom provide guarantees in the worst-case scenario. To address this limitation, Gupta et al. (84) proposed a specially designed CT image reconstruction method that replaces the projector in a projected gradient descent with a CNN and uses the CNN in the feedback loop to recursively project the result onto the sensor domain. This reconstruction method can enforce measurement consistency, is guaranteed to converge, and, under certain conditions, converges to a local minimum of a non-convex inverse problem. On the other hand, while iterative CT reconstruction can yield high-quality images, careful tuning of hyper-parameters in these iterative reconstruction problems is inevitable. To achieve automatic parameter tuning, Shen et al. (143) employed deep reinforcement learning to train a system that can automatically adjust parameters in a human-like manner, and demonstrated that CT images reconstructed from their approach attain quality similar or better than those reconstructed with manually tuned parameters.

For PET, some studies have incorporated specially designed modules to improve the PET image quality. For instance, taking the location-varying contributions from different imaging modalities into account, Wang et al. (67) proposed a locality adaptive fusion module to automatically fuse local patches from multimodal MRI for high-quality PET image synthesis. In Samuel Matej et al. (58), the authors devised a novel Radon inversion layer to address the computational challenges in multi-slice PET image reconstruction. This specially designed layer was demonstrated to be efficient in performing domain transformation from sinogram to image space. Moreover, to encourage feature reuse and prevent resolution degradation, Du et al. (144) designed residual dense connections followed with pixel shuffle operations (RDPS blocks) in the generator network, achieving promising reconstruction results.

### Loss Function

As the task is to restore the quality of the output images in all locations, for the fully supervised learning, the most frequently used loss for the network training is the mean squared error (MSE) between the network prediction and the ground truth. MSE is also known as the L2 loss. Based on MSE, there are also some extended loss functions such as root mean squared errors (RMSE), normalized mean squared errors (NMSE), and normalized root mean squared errors (NRMSE).

There are alternative losses, such as the mean-absolute-error cost function (MAE), which is also known as the L1 loss. Compared with MSE, MAE is used relatively less, but there are still studies showing that using MAE can preserve better results than MSE.

One common choice of loss function for reconstruction problem is L2, but the reconstructed image obtained is of low quality and lacks high-frequency detail. Therefore, in order to offset the shortcoming of L2 loss, structural similarity index (SSIM), signal to noise ratio (SNR), peak SNR (PSNR), or perceptual loss is used as an additional loss to constrain the prediction results in some literatures. These additional loss functions or the combined loss between them have been shown to improve the reconstruction performance of the model.

#### Modality-Specific Loss

In MRI, there are also specially designed losses. In Quan et al. (44), the authors proposed a cyclic data consistency loss, which combines the undersampled frequency loss and the fully reconstructed image loss. In practice, MSE, MAE or other functions can be used as the basic function to achieve cyclic loss. Some studies (52) combine MSE and perceptual loss to form a novel content loss to achieve better reconstruction details. There are also studies that combine MAE with perceptual loss (145), or MSE with TV loss (146), for MR image reconstruction.

In CT, Yang et al. (84) employed for their modified GAN network a joint loss function that combines the Wasserstein distance-based adversarial loss with the well-known pre-trained VGG−19 loss (134). Those two loss terms in the joint loss function are balanced with a hyperparameter to control the trade-off between the GAN adversarial loss and the VGG perceptual loss. When comparing the performance of a modularized deep neural network to commercial algorithms for low-dose CT image reconstruction, Shan et al. (147) chose a composite loss function that includes three components: adversarial loss, MSE, and edge incoherence. The adversarial loss is used to train the generator in their GAN network to produce images as close to the reference high-dose images as possible, the MSE is used to reduce image noise, and the edge incoherence is used to enhance the edge information in the denoised image.

In PET, Kim et al. (68) proposed a novel 3D local linear fitting (LLF) function and incorporated it into the cost function, combining the input image with the DnCNN correcting the unwanted bias and finally enhance the image quality. Similarly, Ouyang et al. (105) designed a GAN model with feature matching technique and task-specific perceptual loss to ensure that the synthesized standard-dose amyloid PET images include the correct features.

### Requirement for Network Training

The fundamental parameter learning schemes are back-propagation algorithms. Adam optimization with variable parameter momentum is often used in neural network optimization. As for hardware, the graphics card for deep learning network training is essential. According to the literature we searched and referenced, the types of graphics cards generally used are NVIDIA K80, NVIDIA K40c, GTX 1080Ti, RTX 2080, RTX 2080Ti, Titan X, Titan Xp, Titan V, etc. As for software, TensorFlow, PyTorch, Keras, Caffe, etc. are several commonly-used DNN training frameworks. In addition, Matlab is also used to process data or perform tests in some studies. The system used is generally a Linux system.

## Clinical Applications and Current Achievements

Deep learning-based medical imaging techniques have played more and more important roles in today's clinical applications, and have achieved significant progress in solving various major pain points in different imaging modalities.

### MRI

**Figure 1:**
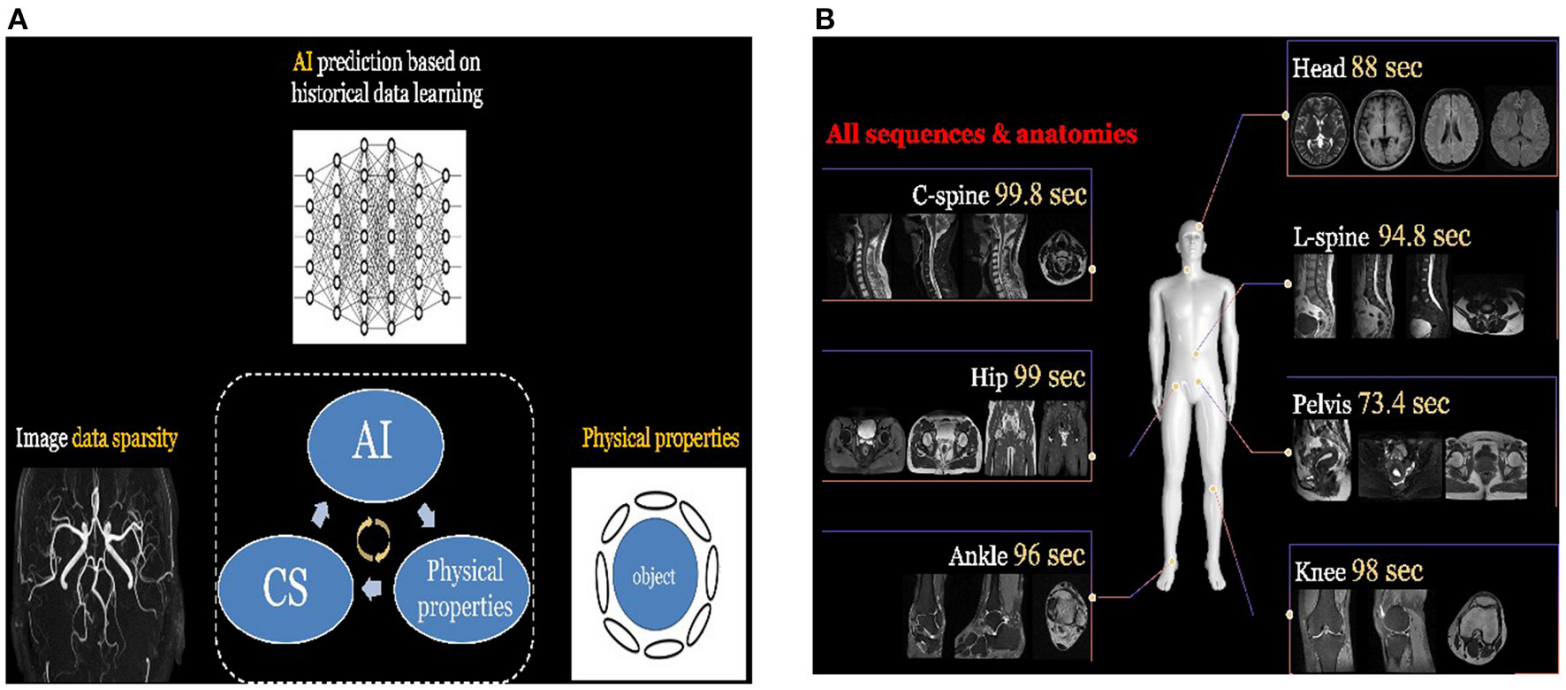
**(A)** The principle of ACS. It integrates the advantages of different acceleration techniques such as deep learning-based reconstruction, parallel imaging, and compressed sensing. **(B)** ACS normally can achieve great scan speed (i.e., <100 s) for different body parts and sequences.

For MRI, it has superior soft-tissue contrast and it is radiation-free. However, the major limitation of MRI is its slow acquisition speed. Although lots of acceleration strategies were proposed in the literature, such as parallel imaging and compressed sensing, they have their own limitations such as amplification of Gibbs artifacts and long iterative reconstruction time. Deep learning-based techniques offer a feasible solution to robustly and efficiently reconstruct the MRI images from subsampled K-space data even under high down sampling factors. Moreover, deep learning-based reconstruction techniques can be integrated with conventional acceleration techniques to reach even higher reconstruction quality. For instance, the AI-assisted compressed sensing (ACS) technique developed by United Imaging Intelligence (UII) and United Imaging Healthcare (UIH) integrates the advantages of four acceleration techniques, i.e., (1) deep learning-based reconstruction, (2) partial Fourier transform, (3) parallel imaging, and (4) compressed sensing, into a unified framework, and achieves great success in real-world clinical applications for fast MRI imaging. ACS is able to reduce around 80% scan time on average for most of the FSE sequences, and it supports the scan of different body parts such as head, cervical spine, lumbar spine, hip, pelvis, ankle, and knee. For each body part, ACS normally can achieve a scan time of fewer than 100 s for all the sequences as shown in Figure 1.

ACS has received FDA 510K clearance and has also been deployed in different hospitals. Another example is the SubtleMR techniques developed by Subtle Medical, which also adopts deep learning-based techniques for fast MR imaging and received FDA 510K clearance. SubtleMR is able to reduce around 60% scan time and has also been deployed in many hospitals and applied in real-world clinical workflow in the US.

### CT

For modalities of CT (as well as PET as introduced below), the radiation dose delivered to the patient must be strictly controlled, because radiation is harmful to the patient and an excessive dose may lead to the result of secondary cancer. However, a lower dose normally leads to inferior image quality, and it may affect the diagnosis accuracy. Therefore, how to obtain high-quality images under the low-dose condition for CT is essential in real world clinical applications.

**Figure 2:**
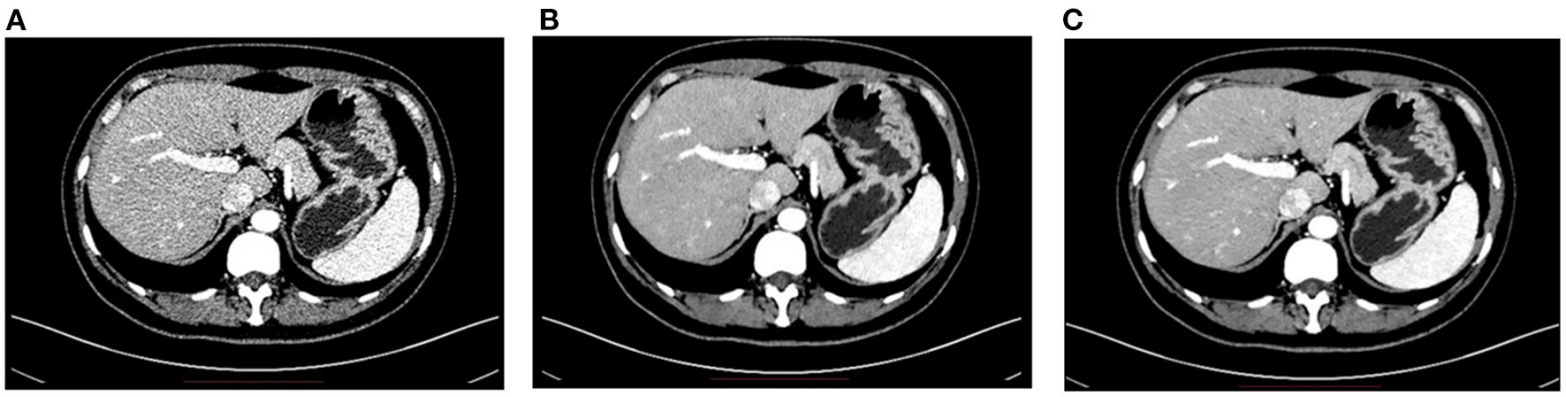
**(A)** The low dose abdominal CT image. **(B)** The resulting image after applying DELTA to the low dose image in **(A)**. **(C)** The corresponding standard dose abdominal CT image.

Deep learning-based denoising techniques provide a good solution to obtain high-quality CT images under low-dose conditions. The basic principle is to train a deep learning network that learns the mapping between the low dose CT image and the corresponding standard-dose CT image. Once the network is trained, the image quality can be significantly improved by passing the low dose CT image through the network. This strategy has been adopted by many industries and turned into products in real world applications. For instance, the DELTA (i.e., DEep Learning Trained Algorithms), a deep learning-based denoising technique developed by UII and UIH, can reduce the dose up to 80% while the low contrast detectability (LCD) of CT images can be improved up to 157%; some typical examples are shown in Figure 2. Canon developed the Advanced intelligent Clear-IQ Engine (AiCE) which can reduce the noise and boost signal in CT images based on deep learning. GE developed the TrueFidelity CT imaging platform, which adopts deep learning-based techniques to improve the image quality of low-dose CT images. DELTA, AiCE, and TrueFidelity all received FDA 510K clearance.

### PET

For PET, besides the concern of dose, another pain point is the relatively longer imaging time than other image modalities such as CT and DR, and some patients such as children and patients with bone cancer may not be able to hold their positions during the imaging process. Therefore, how to obtain high-quality images under low-dose conditions and how to accelerate the imaging is essential for real-world clinical applications of PET.

**Figure 3:**
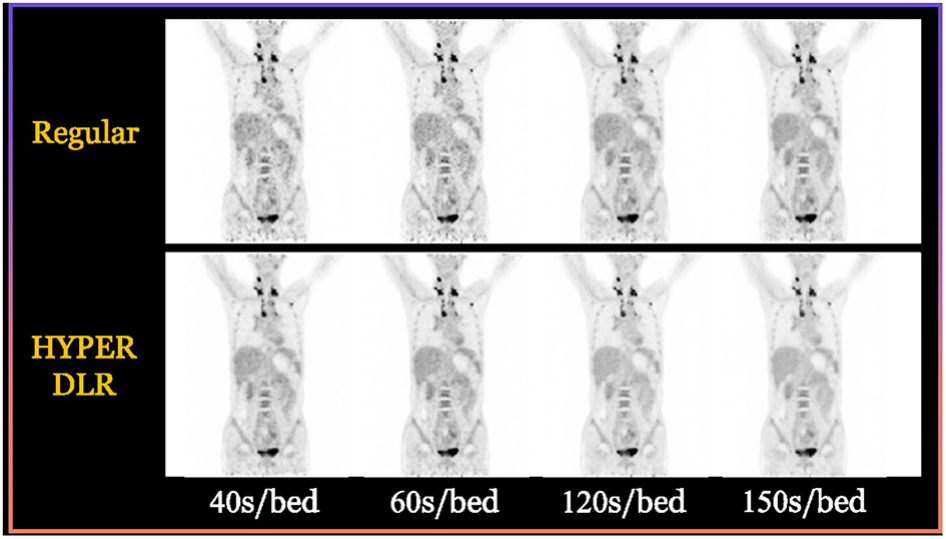
Typical examples of the HYPER DLR PET denoising product developed by UII and UIH. The first row shows PET images obtained by using different acquisition times per bed without HYPER DLR, where the image quality degrades significantly when fast acquisition time. The second row shows the resulting images by applying the HYPER DLR technique, where obvious image quality improvement can be observed.

So far, various deep learning-based techniques have been applied to accelerate the acquisition speed of PET imaging and also maintain the high quality of PET images. For instance, the HYPER DLR (Deep-Learning Reconstruction) product developed by UII and UIH can significantly reduce the scanning time of PET imaging from 3 min/bed to <1 min/bed. In addition, it can effectively reduce the noise level of PET images under low count rate conditions and significantly improve image quality. Specifically, the SNR (Signal-to-Noise Ratios) of PET images can be improved by 42% with an accelerated imaging speed. Figure 3 shows some typical examples of HYPER DLR. Similarly, Subtle Medical developed the SubtlePET product which also adopts deep learning-based techniques and can denoise the low-count PET images obtained in 25% of the original scan duration, improving patient comfort during PET scans. Both HYPER DLR and SubtlePET received FDA 510K clearance.

### PET-MRI

**Figure 4:**
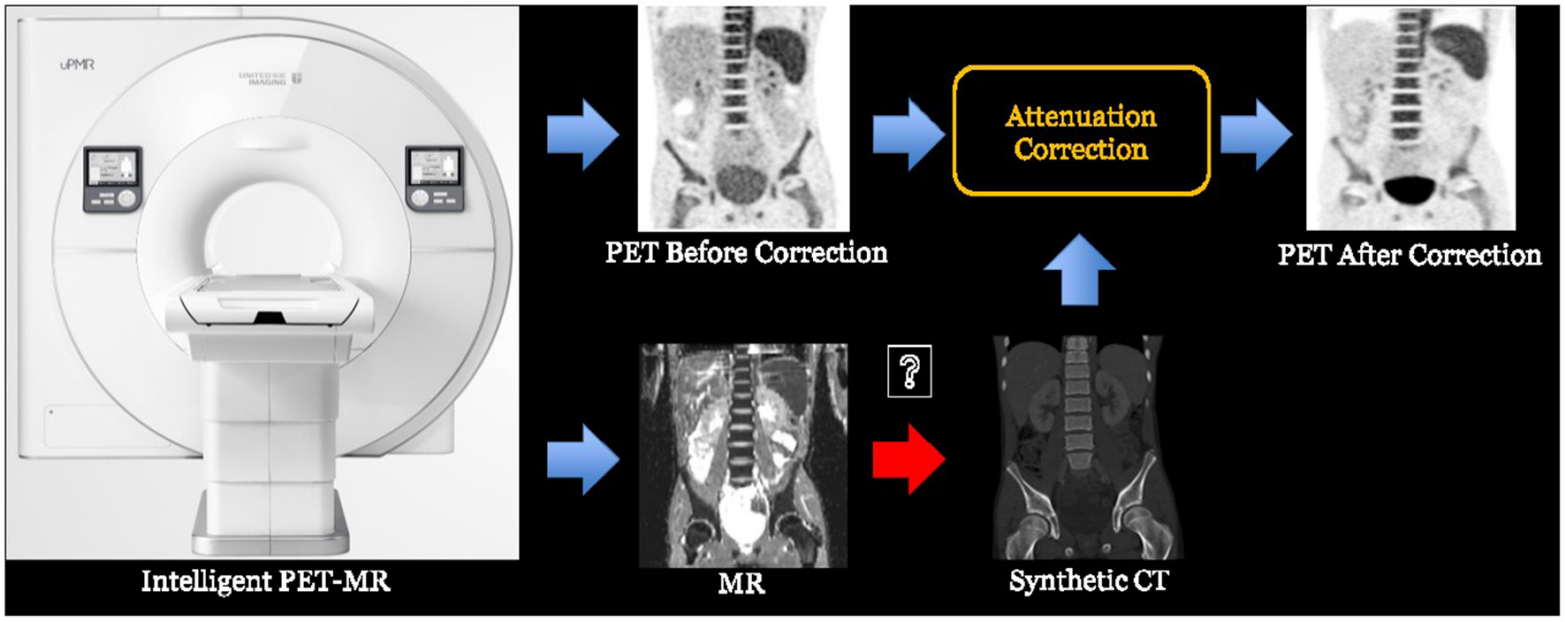
The process of PET-MR attenuation correction, where a synthetic CT image is obtained from the MR image to help the attenuation correction of PET image.

In some applications, cross-modality synthesis techniques are also required. For instance, the PET-MR imaging equipment normally needs to synthesize the CT image from the acquired MR image in order to perform attenuation correction (AC) for the PET image (148). This process is illustrated in Figure 4.

**Figure 5:**
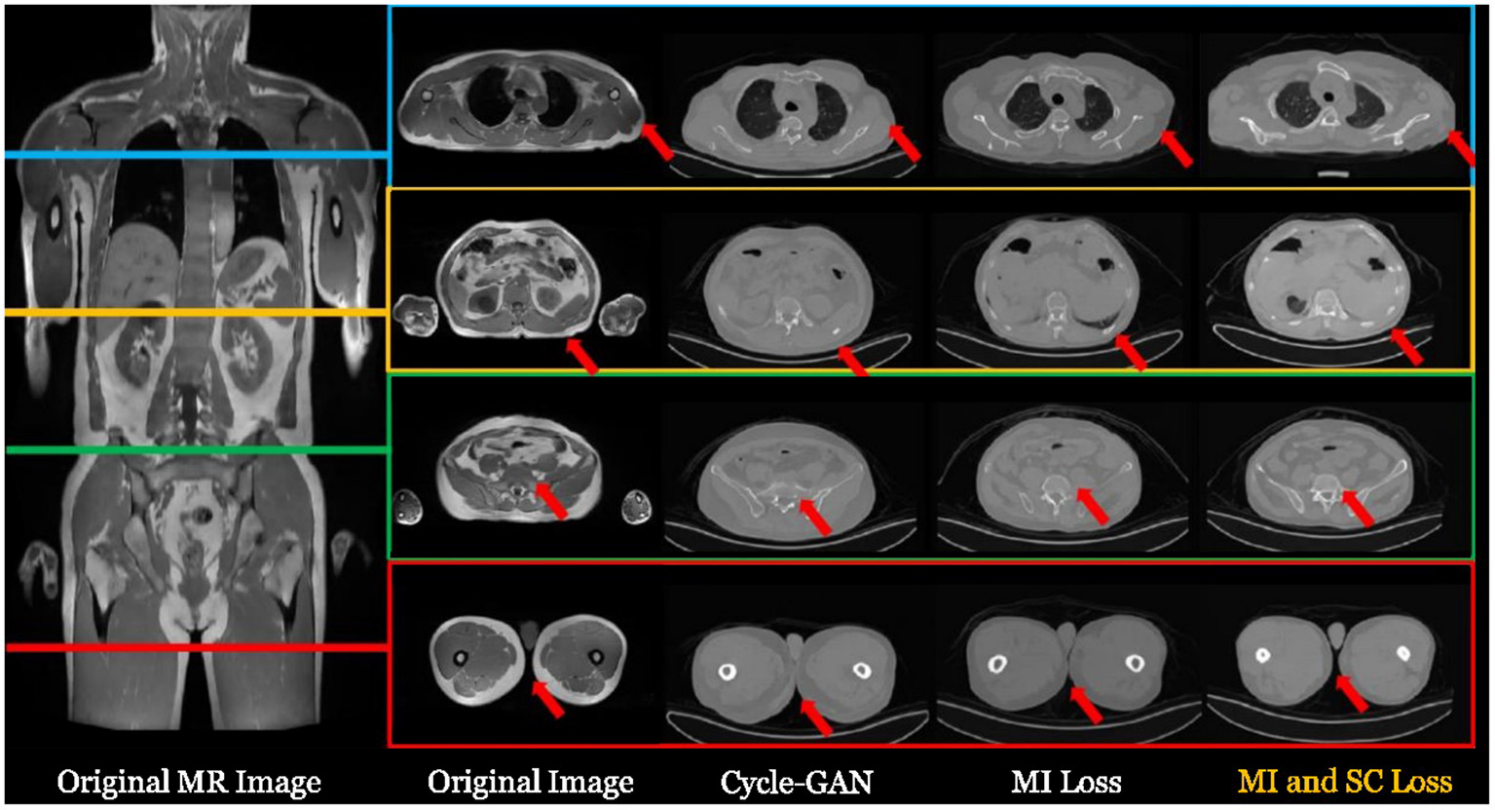
Typical examples of synthesizing CT images from a whole body MR image with deep learning-based techniques. For more details, please refer to Ge et al. (149).

There are lots of synthesis strategies. The most simple and straightforward strategy is to segment the MR image into several tissue types and fill the corresponding regions with fixed CT HU values. This strategy has been widely adopted in many companies such as Siemens and GE. With the aid of deep learning-based cross-modality synthesis techniques, it is possible to obtain more precise synthesized CT images from the MR images with unsupervised learning techniques and therefore to produce more accurate AC operation. For instance, UII and UIH proposed an unsupervised deep learning-based technique (149) that can effectively synthesize the CT images from the MR sequences. Typical examples are shown in Figure 5.

## Challenges and Opportunities

The success of deep learning-based methods on image reconstruction for medical imaging has been extensively validated. However, the wide applications in clinical practices are not yet realized. One key limiting issue is the model interpretability. Due to the nature of DNN, the entire non-linear mapping process is a “black box,” meaning that no direct physical or theoretical mechanism is provided to explain how the inputs are transformed to the outputs (150). Consequently, deep learning reconstruction models find difficulties to get accepted by clinicians. Recently, enhancing model interpretability through building interpretable neural networks or utilizing various visualization techniques becomes a hot topic in deep learning-based natural image analysis (151–154). Similarly, more efforts should be devoted to building both interpretable and high-performance deep learning reconstruction models.

Another challenge is the generalization capability of deep learning-based methods. It is known that deep learning is a data-driven method, and the performance of deep learning models depends heavily on the training data (25, 26, 155). Thus, constructing a comprehensive training dataset is critical. Different from natural images, the distributions of medical images can be quite different if different scanning protocols or scanning machines are utilized. Moreover, due to ethical issues, building large medical image datasets by collecting images from different resources is difficult. As a result, the performance of most existing deep learning models might be over-claimed, and a performance drop can be observed when applying the reported models to the data of end-users. Building robust models that can maintain performance during implementation is important to promote wide applications.

At the same time, the increasing demand for automated image analysis in the clinic to help achieve efficient and accurate imaging-based diagnosis and decision making is providing various opportunities for the introduction of deep learning-based methods. With the rapid development of computing power and optimization of deep learning models, deep learning is expected to play a significant role in achieving fast, portable, safe, and cheap medical imaging. For instance, the transformer (156) framework proposed in 2017 for NLP has demonstrated inspiring performance in capturing global information and has also shown great potential for applications in many image processing tasks recently. The development of the transformer also provides opportunities for the enhancement of current medical imaging models. Besides, multi-modal imaging and autonomous imaging are also promising directions for future studies.

## Conclusion

Deep learning has presented inspiring performances in image reconstruction for different medical imaging modalities, including MRI, CT, and PET. In this review paper, we focus on the applications in MRI, CT, and PET. A detailed survey is conducted in the following aspects and sequence: the overall deep learning reconstruction workflow, the technological development of deep learning reconstruction, the clinical applications and current achievements, and a discussion of the challenges and opportunities. In summary, deep learning-based medical image reconstruction presents a great potential to promote a wide spectrum of applications in the clinic, if the remaining issues, such as interpretability and generalizability, can be properly addressed in the future.

## Author Contributions

SW: MRI review, manuscript preparation, and editing. GC: CT review, manuscript preparation, and editing. YW: PET review, manuscript preparation, and editing. SL, QW, and JS: manuscript preparation and editing. CL: manuscript preparation. DS: topic creating and manuscript editing. All authors contributed to the article and approved the submitted version.

## Funding

This work was supported by National Natural Science Foundation of China (62131015, 62071314, 61871371, and 81830056), Science and Technology Commission of Shanghai Municipality (STCSM) (grant number 21010502600), Sichuan Science and Technology Program (2021YFG0326 and 2020YFG0079), Scientific and Technical Innovation 2030 - New Generation Artificial Intelligence Project (2020AAA0104100 and 2020AAA0104105), Key-Area Research and Development Program of Guangdong Province (2018B010109009), Key Laboratory for Magnetic Resonance and Multimodality Imaging of Guangdong Province (2020B1212060051), the Basic Research Program of Shenzhen (JCYJ20180507182400762), and Youth Innovation Promotion Association Program of the Chinese Academy of Sciences (2019351).

## Conflict of Interest

SL and DS are employed by Shanghai United Imaging Intelligence Co., Ltd. The remaining authors declare that the research was conducted in the absence of any commercial or financial relationships that could be construed as a potential conflict of interest.

## Publisher's Note

All claims expressed in this article are solely those of the authors and do not necessarily represent those of their affiliated organizations, or those of the publisher, the editors and the reviewers. Any product that may be evaluated in this article, or claim that may be made by its manufacturer, is not guaranteed or endorsed by the publisher.

## Footnotes

^1^Popular datasets in MRI include fastMRI https://fastmri.org/dataset/ and brainweb https://brainweb.bic.mni.mcgill.ca/.

## References

[1] Shung KK Smith M Tsui BMW. Principles of Medical Imaging. Academic Press. (2012).

[2] Doi K. Diagnostic imaging over the last 50 years: research and development in medical imaging science and technology. Phys Med Biol. (2006) 51:5–27. 10.1088/0031-9155/51/13/R02 16790920

[3] Westbrook C Talbot J. MRI in Practice. John Wiley & Sons. (2018).

[4] Buzug TM. Computed Tomography. Springer handbook of medical technology. (2011):311–42. 10.1007/978-3-540-74658-4_16

[5] Bénard F Romsa J Hustinx R. Imaging gliomas with positron emission tomography and single-photon emission computed tomography. Semin Nucl Med. (2003) 33:148–62. 10.1053/snuc.2003.127304 12756647

[6] Edelman RR Warach S. Magnetic resonance imaging. N Engl J Med. (1993) 328:785–91. 10.1056/NEJM199303183281109 8369029

[7] Hoult DI Bhakar B. NMR signal reception: Virtual photons and coherent spontaneous emission. Concepts Magn Reson. (1997) 9:277–91. 10.1002/(SICI)1099-0534(1997)9:5<277::AID-CMR1>3.0.CO;2-W11133292

[8] Manduca A Yu L Trzasko JD Khaylova N Kofler JM McCollough CM. Projection space denoising with bilateral filtering and CT noise modeling for dose reduction in CT. Med Phys. (2009) 36:4911–9. 10.1118/1.3232004 19994500 PMC4108640

[9] Gong Y Shan H Teng Y Tu N Liang G Wang G. Parameter-transferred Wasserstein generative adversarial network (PT-WGAN) for low-dose PET image denoising. IEEE Trans Radiat Plasma Med Sci. (2020) 5:213–23. 10.1109/TRPMS.2020.3025071 35402757 PMC8993163

[10] Macovski A Conolly S. Novel approaches to low-cost MRI. Mag Reson Med. (1993) 30:221–30. 10.1002/mrm.1910300211 8366803

[11] Wang S Tan S Gao Y Liu Q Ying L Xiao T. Learning joint-sparse codes for calibration-free parallel MR imaging. IEEE Trans Med Imaging. (2018) 37:251–61. 10.1109/TMI.2017.2746086 28866485

[12] Block KT Uecker M Frahm J. Undersampled radial MRI with multiple coils. Iterative image reconstruction using a total variation constraint. Mag Reson Med. (2007) 57:1086–98. 10.1002/mrm.21236 17534903

[13] Adluru G DiBella EVR. Reordering for improved constrained reconstruction from undersampled k-space data. Int J Biomed Imaging. (2008) 2008:341684. 10.1155/2008/341684 19096715 PMC2603268

[14] Gong K Guan J Kim K Zhang X Yang J Seo Y. Iterative PET image reconstruction using convolutional neural network representation. IEEE Trans Med Imaging. (2018) 38:675–85. 10.1109/TMI.2018.2869871 30222554 PMC6472985

[15] Zhang Z Liang X Dong X Xie Y Cao G A. sparse-view CT reconstruction method based on combination of DenseNet and deconvolution. IEEE Trans Med Imaging. (2018) 37:1407–17. 10.1109/TMI.2018.2823338 29870369

[16] Chartrand R. Fast algorithms for nonconvex compressive sensing: MRI reconstruction from very few data. In: Proceedings of the International Symposium on Biomedical Imaging: From Nano to Macro. Boston (2009). p. 262–5. 10.1109/ISBI.2009.5193034

[17] Lin F Man DB A. hierarchical approach to deep learning and its application to tomographic reconstruction. In: Proceedings of the International Meeting on Fully Three-Dimensional Image Reconstruction in Radiology and Nuclear Medicine Philadelphia. Niskayuna (2019).

[18] Fan J Cao X Wang Q Yapa PT Shen D. Adversarial learning for mono-or multi-modal registration. Med Image Anal. (2019) 58:101545. 10.1016/j.media.2019.101545 31557633 PMC7455790

[19] Cao X Yang J Zhang J Dong N Kim M Wang Q. Deformable image registration based on similarity-steered CNN regression. In: Proceedings of the International Symposium on Medical Image Computing and Computer Assisted Intervention (MICCAI'17). Cham: Springer (2017). 10.1007/978-3-319-66182-7_35 PMC5731783 29250613

[20] Luan H Qi F Xue Z Chen L Shen D. Multimodality image registration by maximization of quantitative-qualitative measure of mutual information. Pattern Recognition, (2008) 41, 285–98. 10.1016/j.patcog.2007.04.002

[21] Zhang J Gao Y Gao Y Munsell BC Shen D. Detecting anatomical landmarks for fast Alzheimer's disease diagnosis. IEEE Trans Med Imaging. (2016) 35:2524–33. 10.1109/TMI.2016.2582386 27333602 PMC5153382

[22] Mohsen H El-Dahshan ESA El-Horbaty ESM Salem ABM. Classification using deep learning neural networks for brain tumors. Future Comput Inform J. (2018) 3:68–71. 10.1016/j.fcij.2017.12.001

[23] Abraham N Khan NM. A novel focal tversky loss function with improved attention u-net for lesion segmentation. In: Proceedings of the International Symposium on Biomedical Imaging (ISBI'19) Venice. (2019). 10.1109/ISBI.2019.875932927295638

[24] Russell SJ Norvig P. Artificial Intelligence: A Modern Approach. 3rd edn. Applied Mechanics & Materials (2009).

[25] Webb S. Deep learning for biology. Nature. (2018) 554:555–7. 10.1038/d41586-018-02174-z 29469107

[26] Lecun Y Bengio Y Hinton G. Deep learning. Nature. (2015) 521:436–44. 10.1038/nature14539 26017442

[27] Shen D Wu G Suk HI. Deep learning in medical image analysis. Annu Rev Biomed Eng. (2017) 19:221–48. 10.1146/annurev-bioeng-071516-044442 28301734 PMC5479722

[28] Murphy KP. Machine learning: a probabilistic perspective. Chance. (2014) 27:62–3. 10.1080/09332480.2014.914768

[29] Hastie T Tibshirani R Friedman J. The Elements of Statistical Learning: Data Mining. Springer Series in Statistics. (2009). 10.1007/978-0-387-84858-7

[30] Mnih V Kavukcuoglu K Silver D Rusu AA Veness J Bellemare MG. Human-level control through deep reinforcement learning. Nature. (2014) 518:529–33. 10.1038/nature14236 25719670

[31] Sutton RS Barto AG. Reinforcement learning: an introduction. Kybernetes. (1998) 27:1093–6. 10.1108/k.1998.27.9.1093.3

[32] Lustig M Donoho DL Santos JM Pauly JM. Compressed sensing MRI. Signal Process Mag. (2008) 25:72–82. 10.1109/MSP.2007.91472827295638

[33] Liang D Liu B Wang J Ying L. Accelerating SENSE using compressed sensing. Magn Reson Med. (2009) 62:1574–84. 10.1002/mrm.22161 19785017

[34] Qu X Guo D Ning B Hou Y Lin Y Cai S. Undersampled MRI reconstruction with patch-based directional wavelets. Magn Reson Imaging. (2012) 30:964–77. 10.1016/j.mri.2012.02.019 22504040

[35] Wang S Su Z Ling L Peng X Zhu S Liang F. Accelerating magnetic resonance imaging via deep learning. In: Proceedings of the International Symposium on Biomedical Imaging (ISBI'16). Prague (2016). 10.1109/ISBI.2016.7493320 PMC6839781 31709031

[36] Han Y Sunwoo L Ye JC. k-space deep learning for accelerated MRI. IEEE Trans Med Imaging. (2020) 39:377–86. 10.1109/TMI.2019.2927101 31283473

[37] Zhu B Liu JZ Cauley SF Rosen BR Rosen MS. Image reconstruction by domain-transform manifold learning. Nature. (2018) 555:87–492. 10.1038/nature25988 29565357

[38] Hammernik K Klatzer T Kobler E Recht MP Sodickson DK Pock T. Learning a variational network for reconstruction of accelerated MRI data. Magn Reson Med. (2018) 79:3055–71. 10.1002/mrm.26977 29115689 PMC5902683

[39] Wang S Xiao T Liu Q Zheng H. Deep learning for fast MR imaging: a review for learning reconstruction from incomplete k-space data. Biomed Signal Process Control. (2021) 68:102579. 10.1016/j.bspc.2021.102579

[40] Yang Y Sun J Li H Xu Z. Deep ADMM-Net for compressive sensing MRI. NIPS. (2017) 10–18.

[41] Zhang J Ghanem B. ISTA-Net: Interpretable optimization-inspired deep network for image compressive sensing. In: Proceedings of the IEEE Conference on Computer Vision and Pattern Recognition (CVPR'18). Salt Lake (2018). p. 1828–37. 10.1109/CVPR.2018.00196

[42] Adler J Öktem O. Solving ill-posed inverse problems using iterative deep neural networks. Inverse Probl. (2017) 33:124007. 10.1088/1361-6420/aa9581

[43] Wang S Cheng H Ying L Xiao T Ke Z Zheng H. Deep complex MRI: exploiting deep residual network for fast parallel MR imaging with complex convolution. Magn Reson Imaging. (2020) 68:136–47. 10.1016/j.mri.2020.02.002 32045635

[44] Quan TM Nguyen-Duc T Jeong WK. Compressed sensing MRI reconstruction using a generative adversarial network with a cyclic loss. IEEE Trans Med Imaging. (2018) 37:1488–97. 10.1109/TMI.2018.2820120 29870376

[45] Feldkamp LA Davis LC Kress JW. Practical cone-beam algorithm. J Opt Soc Am. (1984) 1:612–9. 10.1364/JOSAA.1.000612

[46] Gordon R Bender R Herman GT. Algebraic reconstruction techniques (ART) for three-dimensional electron microscopy and X-ray photography. J Theor Biol. (1970) 29:477–81. 10.1016/0022-5193(70)90109-8 5492997

[47] Andersen AH Kak AC. Simultaneous algebraic reconstruction technique (SART): a superior implementation of the ART algorithm. Ultrason Imaging. (1984) 6:81–94. 10.1177/016173468400600107 6548059

[48] Erdogan H Fessler JA. Ordered subsets algorithms for transmission tomography. Phys Med Biol. (1999) 44:2835–51. 10.1088/0031-9155/44/11/311 10588288

[49] Lange K Carson R. EM reconstruction algorithms for emission and transmission tomography. J Comput Assist Tomogr. (1984) 8:306–16. 10.1097/00004728-198404000-00002 6608535

[50] Thibault JB Sauer KD Bouman CA Hsieh J. A three-dimensional statistical approach to improved image quality for multislice helical CT. Med Phys. (2007) 34:4526–44. 10.1118/1.2789499 18072519

[51] Zhang Z Yu S Qin W Liang X Xie Y Cao G. Self-supervised CT super-resolution with hybrid model. Comput Biol Med. (2021) 138:104775. 10.1016/j.compbiomed.2021.104775 34666243

[52] Yang G Yu S Dong H Slabaugh G Dragotti PL Ye X. DAGAN: deep De-aliasing generative adversarial networks for fast compressed sensing MRI reconstruction. IEEE Trans Med Imaging. (2018) 37:1310–21. 10.1109/TMI.2017.2785879 29870361

[53] Kang E Min J Ye JC. A deep convolutional neural network using directional wavelets for low-dose X-ray CT reconstruction. Med Phys. (2016) 44:e360–75. 10.1002/mp.12344 29027238

[54] Chen H Zhang Y Kalra NK Lin F Chen Y Liao P. Low-dose CT with a residual encoder-decoder convolutional neural network. IEEE Trans Med Imaging. (2017) 36:2524–35. 10.1109/TMI.2017.2715284 28622671 PMC5727581

[55] Zhang C Xing Y. CT artifact reduction via U-net CNN. In: Proceedings of the Medical Imaging 2018: Image Processing (2018). 10.1117/12.229390317645476

[56] Han Y Ye JC. Framing U-Net via deep convolutional framelets: application to sparse-view CT. IEEE Trans Med Imaging. (2018) 37:1418–29. 10.1109/TMI.2018.2823768 29870370

[57] Park J Hwang D YunKim K Kang S Kim YK Lee JS. Computed tomography super-resolution using deep convolutional neural network. Phys Med Biol. (2018) 63:145011. 10.1088/1361-6560/aacdd4 29923839

[58] Samuel Matej S Daube-Witherspoon ME Karp JS. Analytic TOF PET reconstruction algorithm within DIRECT data partitioning framework. Phys Med Biol. (2016) 61:3365–86. 10.1088/0031-9155/61/9/3365 27032968 PMC5084694

[59] Defrise M Kinahan P. Data Acquisition and Image Reconstruction for 3d33d Pet. Springer (1998). p. 11–53. 10.1007/978-94-017-3475-2_2

[60] Shepp LA Vardi Y. Maximum likelihood reconstruction for emission tomography. IEEE Trans Med Imaging. (1982) 1:113–22. 10.1109/TMI.1982.4307558 18238264

[61] Hudson HM Larkin RS. Accelerated image reconstruction using ordered subsets of projection data. IEEE Trans Med Imaging. (1994) 13:601–9. 10.1109/42.363108 18218538

[62] Kang J Gao Y Shi F Lalush DS Lin W Shen D. Prediction of standard-dose brain PET image by using MRI and low-dose brain [18F]FDG PET images. Med Phys. (2015) 42:5301–9. 10.1118/1.4928400 26328979 PMC4545052

[63] Wang Y Zhang P An L Ma G Kang J Shi F. Predicting standard-dose PET image from low-dose PET and multimodal MR images using mapping-based sparse representation. Phys Med Biol. (2016) 61:791–812. 10.1088/0031-9155/61/2/791 26732849

[64] An L Zhang P Adeli E Wang Y Ma G Shi F. Multi-level canonical correlation analysis for standard-dose PET image estimation. IEEE Trans Image Process. (2016) 25:3303–15. 10.1109/TIP.2016.2567072 27187957 PMC5106345

[65] Xiang L Qiao Y Nie D An L Lin W Wang Q. Deep auto-context convolutional neural networks for standard-dose PET image estimation from low-dose PET/MRI. Neurocomputing. (2017) 267:406–16. 10.1016/j.neucom.2017.06.048 29217875 PMC5714510

[66] Xu J Gong E Pauly J Zaharchuk G. 200x Low-dose PET reconstruction using deep learning. (2017). arXiv:1712.04119.

[67] Wang Y Zhou L Yu B Wang L Zu C Lalush DS. 3D auto-context-based locality adaptive multi-modality GANs for PET synthesis. IEEE Trans Med Imaging. (2019) 38:1328–39. 10.1109/TMI.2018.2884053 30507527 PMC6541547

[68] Kim K Wu D Gong K Dutta J Kim JH Son Y. Penalized PET reconstruction using deep learning prior and local linear fitting. IEEE Trans Med Imaging. (2018) 37:1478–87. 10.1109/TMI.2018.2832613 29870375 PMC6375088

[69] Gong K Catana C Qi J Li Q. PET image reconstruction using deep image prior. IEEE Trans Med Imaging. (2019) 38:1655–65. 10.1109/TMI.2018.2888491 30575530 PMC6584077

[70] Feng Q Liu H. Rethinking PET image reconstruction: ultra-low-dose, sinogram and deep learning. In: Proceedings of the International Conference on Medical Image Computing and Computer Assisted Intervention (MICCAI'20) (2020). p. 783–92. 10.1007/978-3-030-59728-3_76

[71] Liu Z Chen H Liu H. Deep learning based framework for direct reconstruction of PET images. In: Proceedings of the International Conference on Medical Image Computing and Computer Assisted Intervention (MICCAI'19). Springer (2019). p. 48–56. 10.1007/978-3-030-32248-9_6

[72] Zhou L Schaefferkoetter JD Tham IWK Gang H Yan J. Supervised learning with CycleGAN for low-dose FDG PET image denoising. Med Image Anal. (2020) 65:101770. 10.1016/j.media.2020.101770 32674043

[73] Huang Q Xian Y Yang D Qu H Yi J Wu P. Dynamic MRI reconstruction with end-to-end motion-guided network. Med Image Anal. (2020) 68:1010901. 10.1016/j.media.2020.101901 33285480

[74] Wang S Ke Z Cheng H Jia S Ying L Zheng H. DIMENSION: dynamic MR imaging with both k-space and spatial prior knowledge obtained via multi-supervised network training. NMR Biomed. (2019) 1–16. 10.1002/nbm.4131 31482598

[75] Aggarwal HK Mani MP Jacob M. MoDL: Model-based deep learning architecture for inverse problems. IEEE Trans Med Imaging. (2019) 38:394–405. 10.1109/TMI.2018.2865356 30106719 PMC6760673

[76] Chen Y Xiao T Li C Liu Q Wang S. Model-based convolutional de-aliasing network learning for parallel MR imaging. In: Proceedings of the International Conference on Medical Image Computing and Computer Assisted Intervention (MICCAI'19). Springer (2019). p. 30–8. 10.1007/978-3-030-32248-9_4

[77] Yaman B Hosseini SAH Moeller S Ellermann J Ugurbil K Akçakaya M. Self-supervised physics-based deep learning MRI reconstruction without fully-sampled data. In: Proceedings of the International Symposium on Biomedical Imaging (ISBI'20). Iowa City (2020). 10.1109/ISBI45749.2020.909851427295638

[78] Hu Y Xu Y Tian Q Chen F Shi X Moran CJ. RUN-UP: accelerated multishot diffusion-weighted MRI reconstruction using an unrolled network with U-net as priors. Magn Reson Med. (2020) 85:709–20. 10.1002/mrm.28446 32783339 PMC8095163

[79] Kang E Cahng W Yoo J Ye JC. Deep convolutional framelet denosing for low-dose CT via wavelet residual network. IEEE Trans Med Imaging. (2018) 37:1358–69. 10.1109/TMI.2018.2823756 29870365

[80] Hwan K McCann MT Froustey E Unser M. Deep convolutional neural network for inverse problems in imaging. IEEE Trans Image Process. (2017) 26:4509–22. 10.1109/TIP.2017.2713099 28641250

[81] Wolterink JM Leiner T Viergever MA Išgum I. Generative adversarial networks for noise reduction in low-dose CT. IEEE Trans Med Imaging. (2017) 36:2536–45. 10.1109/TMI.2017.2708987 28574346

[82] Yang Q Yan P Zhang Y Yu H Shi Y Mou X. Low-dose CT image denoising using a generative adversarial network with wasserstein distance and perceptual loss. IEEE Trans Med Imaging. (2018) 37:1348–57. 10.1109/TMI.2018.2827462 29870364 PMC6021013

[83] You C Li G Zhang Y Zhang X Shan H Li M. CT super-resolution GAN constrained by the identical, residual, and cycle learning ensemble (GAN-CIRCLE). IEEE Trans Med Imaging. (2020) 39:188–203. 10.1109/TMI.2019.2922960 31217097 PMC11662229

[84] Gupta H Jin KH Nguyen HQ McCann MT Unser M. CNN-based projected gradient descent for consistent CT image reconstruction. IEEE Trans Med Imaging. (2018) 37:1440–53. 10.1109/TMI.2018.2832656 29870372

[85] Xiang L Wang L Gong E Zaharchuk G Zhang T. Noise-aware standard-dose PET reconstruction using general and adaptive robust loss. In: Proceedings of the International Conference on Machine Learning in Medical Imaging (MLMI'20). (2020). p. 654–62. 10.1007/978-3-030-59861-7_66

[86] Bevilacqua A Bollini D Campanini R Lanconelli N Galli M. A new approach to image reconstruction in positron emission tomography using artificial neural networks. Int J Mod Phys C. (1998) 9:71–85. 10.1142/S012918319800007830780137

[87] Yang B Ying L Tang J. Artificial neural network enhanced Bayesian PET image reconstruction. IEEE Trans Med Imaging. (2018) 37:1297–309. 10.1109/TMI.2018.2803681 29870360 PMC6132251

[88] Spuhler K Serrano-Sosa1a M Cattell R DeLorenzo C Huang C. Full-count PET recovery from low-countimage using a dilated convolutional neural network. Med Phys. (2020) 47:4928–38. 10.1002/mp.14402 32687608

[89] Whiteley W Luk WK Gregor J DirectPET: full-size neural network PET reconstruction from sinogram data. J Med Imag. (2020) 7: 032503. 10.1117/1.JMI.7.3.032503 32206686 PMC7048204

[90] Xie Z Li T Zhang X Qi W Asma E Qi J. Anatomically aided PET image reconstruction using deep neural networks. Med Phys. (2021) 48:5244–58. 10.1002/mp.15051 34129690 PMC8510002

[91] Sandino CM Lai P Vasanawala SS Cheng JY. Accelerating cardiac cine MRI using a deep learning-based ESPIRiT reconstruction. Magn Reson Med. (2021) 85:152–67. 10.1002/mrm.28420 32697891 PMC7722220

[92] Lee D Yoo J Ye JC. Deep residual learning for compressed sensing MRI. In: Proceedings of the International Symposium on Biomedical Imaging (ISBI'17). Melbourne (2017). p. 15–8. 10.1109/ISBI.2017.7950457

[93] Ke Z Cheng J Ying L Zheng H Zhu Y Liang D. An unsupervised deep learning method for multi-coil cine *MRI*. Phys Med Biol. (2020) 65:235041. 10.1088/1361-6560/abaffa 33263316

[94] Liu X Zhang M Liu Q Xiao T Zheng H Ying L. Multi-contrast MR reconstruction with enhanced denoising autoencoder prior learning. In: Proceedings of the International Symposium on Biomedical Imaging (ISBI'20). Iowa (2020). p. 1432–6. 10.1109/ISBI45749.2020.9098334

[95] Mills AF Sakai O Anderson SW Jara H. Principles of quantitative MR imaging with illustrated review of applicable modular pulse diagrams. Radiographics. (2017) 37:2083–105. 10.1148/rg.2017160099 28985137

[96] Kwee TC Kwee RM. Chest CT in COVID-19: What the radiologist needs to know. Radiographics. (2020) 40:1848–65. 10.1148/rg.2020200159 33095680 PMC7587296

[97] Vayá MDLI Saborit JM Montell JA Pertusa A Bustos A Cazorla M. BIMCV COVID-19+: a large annotated dataset of RX and CT images from COVID-19 patients. (2020). arXiv:2006.01174.

[98] Wang H Xu Y Zhao Y Zhao Y. A novel static PET image reconstruction method. In: Proceedings of the Chinese Automation Congress (CAC'17). Jinan (2017). p. 4537–41. 10.1109/CAC.2017.8243580

[99] Serrano-Sosa M Spuhler K DeLorenzo C Huang C. PET image denoising using structural MRI with a novel dilated convolutional neural network. J Nucl Med. (2020) 61:434. 10.1002/mp.14402 32687608

[100] Ladefoged CN Hasbak P Hornnes C Højgaard L Andersen FL. Low-dose PET image noise reduction using deep learning: application to cardiac viability FDG imaging in patients with ischemic heart disease. Phys Med Biol. (2021) 66:054003. 10.1088/1361-6560/abe225 33524958

[101] Novosad P Reader AJ. MR-guided dynamic PET reconstruction with the kernel method and spectral temporal basis functions. Phys Med Biol. (2016) 61:4624–45. 10.1088/0031-9155/61/12/4624 27227517

[102] Cui J Liu X Wang Y Liu H. Deep reconstruction model for dynamic PET images. PLoS ONE. (2017) 12:e0184667. 10.1371/journal.pone.0184667 28934254 PMC5608245

[103] Gong K Guan J Liu CC Qi J. PET image denoising using a deep neural network through fine tuning. IEEE Trans Radiat Plasma Med Sci. (2018) 3:153–61. 10.1109/TRPMS.2018.2877644 32754674 PMC7402614

[104] Sun H Peng L Zhang H He Y Cao S Lu L. Dynamic PET image denoising using deep image prior combined with regularization by denoising. IEEE Access. (2021) 9:52378–92. 10.1109/ACCESS.2021.306923627295638

[105] Ouyang J Chen KT Gong E Pauly J Zaharchuk G. Ultra-low-dose PET reconstruction using generative adversarial network with feature matching and task-specific perceptual loss. Med Phys. (2019) 46:3555–64. 10.1002/mp.13626 31131901 PMC6692211

[106] Xu J Liu H. Three-dimensional convolutional neural networks for simultaneous dual-tracer PET imaging. Phys Med Biol. (2019) 64:185016. 10.1088/1361-6560/ab3103 31292287

[107] Xu J Liu H. Hybrid-loss guided 3D CNN for dynamic dual-tracer PET reconstruction. In: Proceedings of the International Symposium on Biomedical Imaging (ISBI'19). Venice (2019).

[108] Ellis S Mallia A McGinnity CJ Cook GJR reader AJ. Guided image reconstruction for multi-tracer PET. In: Proceedings of the Nuclear Science Symposium and Medical Imaging Conference (NSS/MIC'17). Atlanta (2017). 10.1109/NSSMIC.2017.8533140

[109] Hong X Zan Y Weng F Tao W Peng Q Huang Q. Enhancing the image quality via transferred deep residual learning of coarse PET sinograms. IEEE Trans Med Imaging. (2018) 37:2322–32. 10.1109/TMI.2018.2830381 29993685

[110] Häggström I Schmidtlein C R Campanella G Fuchs TJ. DeepPET: a deep encoder–decoder network for directly solving the PET image reconstruction inverse problem. Med Image Anal. (2019) 54:253–62. 10.1016/j.media.2019.03.013 30954852 PMC6537887

[111] Kwon K Kim D Park H. A parallel MR imaging method using multilayer perceptron. Med Phys. (2017) 44:6209–24. 10.1002/mp.12600 28944971

[112] Ronneberger O Fischer P Brox T. U-Net: Convolutional networks for biomedical image segmentation. In: Proceedings of the International Conference on Medical Image Computing and Computer Assisted Intervention (MICCAI'15). Springer (2015). p. 234–41. 10.1007/978-3-319-24574-4_28

[113] Falk T Mai D Bensch R Çiçek Ö Abdulkadir A Marrakchi Y. U-Net: deep learning for cell counting, detection, and morphometry. Nat Methods. (2019) 16:67–70. 10.1038/s41592-018-0261-2 30559429

[114] Lee D Yoo J Tak S Ye JC. Deep residual learning for accelerated MRI using magnitude and phase networks. IEEE Trans Biomed Eng. (2018) 65:1985–95. 10.1109/TBME.2018.2821699 29993390

[115] Han Y Yoo J Kim HH Shin HJ Sung K Ye JC. Deep learning with domain adaptation for accelerated projection-reconstruction MR. Magn Reson Med. (2018) 80:1189–205. 10.1002/mrm.27106 29399869

[116] Duan C Deng H Xiao S Xie J Li H Sun X. Fast and accurate reconstruction of human lung gas MRI with deep learning. Magn Reson Med. (2019) 82:2273–85. 10.1002/mrm.27889 31322298

[117] Hyun CM Kim HP Lee SM Lee S Seo JK. Deep learning for undersampled MRI reconstruction. Phys Med Biol. (2018) 63:135007. 10.1088/1361-6560/aac71a 29787383

[118] Kofler A Haltmeier M Kolbitsch C Kachelrieß M Dewey M. A U-Nets cascade for sparse view computed tomography. In: Proceedings of the Machine Learning for Medical Image Reconstruction (MLMIR'18). Springer (2018). p. 91–9. 10.1007/978-3-030-00129-2_11

[119] Liu Y Zhang Y. Low-dose CT restoration via stacked sparse denoising autoencoders. Neurocomputing. (2018) 284:80–9. 10.1016/j.neucom.2018.01.015

[120] Schaefferkoetter J Yan J Ortega C Sertic A Lechtman E Eshet Y. Convolutional neural networks for improving image quality with noisy PET data. EJNMMI Res. (2020) 10:105. 10.1186/s13550-020-00695-1 32955669 PMC7505915

[121] Sikka A Peri SV Bathula DR. MRI to FDG-PET: cross-modal synthesis using 3D U-Net for multi-modal Alzheimer's classification. In: Proceedings of the International Conference on Simulation and Synthesis in Medical Imaging (SASHIMI'18). Springer (2018). 10.1007/978-3-030-00536-8_9

[122] Cui J Gong K Guo N Wu C Meng X Kim K. PET image denoising using unsupervised deep learning. Eur J Nucl Med Mol Imaging. (2019) 46:2780–9. 10.1007/s00259-019-04468-4 31468181 PMC7814987

[123] He K Zhang X Ren S Sun J. Deep residual learning for image recognition. In: Proceedings of the International Conference on Computer Vision and Pattern Recognition (CVPR'16). Las Vegas (2016). p. 770–8.

[124] Shi J Liu Q Wang C Zhang Q Ying S Xu H. Super-resolution reconstruction of MR image with a novel residual learning network algorithm. Phys Med Biol. (2018) 63:085011. 10.1088/1361-6560/aab9e9 29583134

[125] Shi J Li Z Ying S Wang C Zhang Q Yan P. image super-resolution via wide residual networks with fixed skip connection. IEEE J Biomed Health Inform. (2019) 23:1129–40. 10.1109/JBHI.2018.2843819 29993565

[126] Li X Cao T Tong Y Ma X Niu Z Guo H. Deep residual network for highly accelerated fMRI reconstruction using variable density spiral trajectory. Neurocomputing. (2020) 398:338–46. 10.1016/j.neucom.2019.02.070

[127] Du J He Z Wang L Gholipour A Zhou Z Chen D. Super-resolution reconstruction of single anisotropic 3D MR images using residual convolutional neural network. Neurocomputing. (2020) 392:209–20. 10.1016/j.neucom.2018.10.102

[128] Chen KT Gong E de Carvalho Macruz FB Xu J Boumis A Khalighi M. Ultra–low-dose 18F-florbetaben amyloid PET imaging using deep learning with multi-contrast MRI inputs. Radiology. (2019) 290:649–56. 10.1148/radiol.2018180940 30526350 PMC6394782

[129] Dong X Vekhande S Cao G. Sinogram interpolation for sparse-view micro-CT with deep learning neural network. In: Proceedings of the Medical Imaging 2019: Physics of Medical Imaging (2019).

[130] Goodfellow IJ Pouget-Abadie J Mirza M Xu B Warde-Farley D Ozair S. Generative adversarial nets. Adv Neural Inform Processing Syst. (2014) 2672–80.

[131] Mardani M Gong E Cheng JY Vasanawala SS Zaharchuk G Xing L. Deep generative adversarial neural networks for compressive sensing MRI. IEEE Trans Med Imaging. (2019) 38:167–79. 10.1109/TMI.2018.2858752 30040634 PMC6542360

[132] Simonyan K Zisserman A. Very deep convolutional networks for large-scale image recognition. (2014). arXiv:1409.1556.

[133] Wang Y Yu B Wang L Zu C Lalush DS Lin W. 3D conditional generative adversarial networks for high-quality PET image estimation at low dose. Neuroimage. (2018) 174:550–62. 10.1016/j.neuroimage.2018.03.045 29571715 PMC6410574

[134] Ben-Cohen A Klang E Raskin SP Soffer S Ben-Haim S Kone E.. Cross-modality synthesis from CT to PET using FCN and GAN networks for improved automated lesion detection. Eng Appl Artif Intell. (2019) 78:186–94. 10.1016/j.engappai.2018.11.013

[135] Yaakub SN McGinnity CJ Clough JR Kerfoot E Girard N Guedj E. Pseudo-normal PET synthesis with generative adversarial networks for localising hypometabolism in epilepsies. In: Proceedings of the International Workshop on Simulation and Synthesis in Medical Imaging. (2019). 10.1007/978-3-030-32778-1_5

[136] Kaplan S Zhu Y-M. Full-dose PET image estimation from low-dose PET image using deep learning: a pilot study. J Digit Imaging. (2019) 32:773–8. 10.1007/s10278-018-0150-3 30402670 PMC6737135

[137] Yang L Dong X Wang T Higgins K Liu T Curran WJ. Whole-body PET estimation from low count statistics using cycle-consistent generative adversarial networks. Phys Med Biol. (2019) 64:215017. 10.1088/1361-6560/ab4891 31561244 PMC7764437

[138] Schlemper J Caballero J Hajnal JV Price AN Rueckert D A. deep cascade of convolutional neural networks for dynamic MR image reconstruction. IEEE Trans Med Imaging. (2018) 37:491–503. 10.1109/TMI.2017.2760978 29035212

[139] Qin C Schlemper J Caballero J Price AN Hajnal JV Rueckert D. Convolutional recurrent neural networks for dynamic MR image reconstruction. IEEE Trans Med Imaging. (2019) 38:280–90. 10.1109/TMI.2018.2863670 30080145

[140] Ran M Xia W Huang Y Lu Z Bao P Liu Y. MD-Recon-Net: a parallel dual-domain convolutional neural network for compressed sensing MRI. IEEE Trans Radiat Plasma Med Sci. (2020) 5:120–35. 10.1109/TRPMS.2020.299187727295638

[141] El-Rewaidy H Neisius U Mancio J Kucukseymen S Rodriguez J Paskavitz A. Deep complex convolutional network for fast reconstruction of 3D late gadolinium enhancement cardiac MRI. NMR Biomed. (2020) 33:e4312. 10.1002/nbm.4312 32352197

[142] Cole E Cheng J Pauly J Vasanawala S. Analysis of deep complex-valued convolutional neural networks for MRI reconstruction and phase-focused applications. Magn Reson Med. (2021) 86:1093–109. 10.1002/mrm.28733 33724507 PMC8291740

[143] Shen C Gonzalez Y Chen L Jiang SB Jia X. Intelligent parameter tuning in optimization-based iterative CT reconstruction via deep reinforcement learning. IEEE Trans Med Imaging. (2018) 37:1430–9. 10.1109/TMI.2018.2823679 29870371 PMC5999035

[144] Du Q Qiang Y Yang W Wang Y Muhhammad BZ. DRGAN: a deep residual generative adversarial network for PET image reconstruction. IET Image Processing. (2020) 14:1690–700. 10.1049/iet-ipr.2019.1107

[145] Huang Q Yang D Wu P Qu H Yi J Metaxas D. reconstruction via cascaded channel-wise attention network. In: Proceedings of the International Symposium on Biomedical Imaging (ISBI'19). Venice (2019). 10.1109/ISBI.2019.8759423

[146] Zhang T Jackson1 LH Uus A Clough JR Story L Rutherford MA. Self-supervised recurrent neural network for 4D abdominal and In-utero MR imaging. In: International Workshop on Machine Learning for Medical Image Reconstruction. Springer (2019). 10.1007/978-3-030-33843-5_2

[147] Shan H Padole A Homayounieh F Kruger U Khera RD Nitiwarangkul C. Competitive performance of a modularized deep neural network compared to commercial algorithms for low-dose CT image reconstruction. Nat Mach Intell. (2019) 1:269–76. 10.1038/s42256-019-0057-9 33244514 PMC7687920

[148] Xiang L Wang Q Nie D Zhang L Jin X Qiao Y. Deep embedding convolutional neural network for synthesizing CT image from T1-Weighted MR image. Med Image Anal. (2018) 47:31–44. 10.1016/j.media.2018.03.011 29674235 PMC6410565

[149] Ge Y Wei D Xue Z Wang Q Zhou X Zhan Y. Unpaired Mr to CT synthesis with explicit structural constrained adversarial learning. In: Proceedings of the International Symposium on Biomedical Imaging (ISBI'19). Venice (2019). p. 1096–99.

[150] Koh PW Liang P. Understanding black-box predictions via influence functions. In: Proceedings of the International Conference on Machine Learning (PMLR'17). Sydney (2017). p. 2976–87.

[151] Zhang Q Wu Y Zhu S-C. Interpretable convolutional neural networks. In: Proceedings of the International Conference on Computer Vision and Pattern Recognition (CVPR'18). Salt Lake (2018). p. 8827–36. 10.1109/CVPR.2018.00920

[152] Pope PE Kolouri S Rostami M Martin CE Hoffmann H. Explainability methods for graph convolutional neural networks. In: Proceedings of the International Conference on Computer Vision and Pattern Recognition (CVPR'19). Long Beach (2019). p. 10772–81. 10.1109/CVPR.2019.01103

[153] Zhou B Khosla A Lapedriza A Oliva A Torralba A. Learning deep features for discriminative localization. In: Proceedings of the International Conference on Computer Vision and Pattern Recognition (CVPR'16). Las Vegas (2016). p. 2921–9. 10.1109/CVPR.2016.319

[154] Selvaraju RR Cogswell M Das A Vedantam R Parikh D Batra D. Grad-CAM: visual explanations from deep networks via gradient-based localization. In: Proceedings of the International Conference on Computer Vision and Pattern Recognition (CVPR'17). Springer (2017). p. 618–26. 10.1109/ICCV.2017.74

[155] Towards trustable machine learning. Nat Biomed Eng. (2018) 2:709–10. 10.1038/s41551-018-0315-x 31015650

[156] Vaswani A Shazeer N Parmar N Uszkoreit J Jones L Gomez AN. Attention is all you need. (2017). arXiv:1706.03762.

